# Which Way? Quit Pack study protocol: a national hybrid type 2 effectiveness-implementation study of mailed smoking and vaping cessation support for Aboriginal and Torres Strait Islander people

**DOI:** 10.1136/bmjopen-2026-121440

**Published:** 2026-09-11

**Authors:** Kade Booth, Jamie Bryant, Kayden Roberts-Barker, Zabowie Mills, Tanika Ridgeway, Raymond Kelly, Felicity Collis, Billie Bonevski, Raglan Maddox, Lisa J Whop, Catherine Chamberlain, Alexandra L C Martiniuk, Mary Belfrage, Cathy Segan, Christopher Doran, Christopher Oldmeadow, Lucy Leigh, Michelle Kennedy

**Affiliations:** 1 The University of Newcastle, Callaghan, New South Wales, Australia; 2 Hunter Medical Research Institute, New Castle, New South Wales, Australia; 3 School of Medicine and Public Health, The University of Newcastle, Waratah, New South Wales, Australia; 4 Health Behaviour Research Group, Priority Research Centre for Health Behaviour, Hunter Medical Research Institute, Newcastle, New South Wales, Australia; 5 Flinders University College of Medicine and Public Health, Bedford Park, South Australia, Australia; 6 National Centre for Aboriginal and Torres Strait Islander Wellbeing Research–Yardhura Walani, Australian National University, Canberra, Australian Capital Territory, Australia; 7 Melbourne School of Population and Global Health, University of Melbourne, Melbourne, Victoria, Australia; 8 University of Sydney, Sydney, New South Wales, Australia; 9 International Centre for Future Health Systems, University of New South Wales, Sydney, New South Wales, Australia; 10 University of Melbourne, Melbourne, Victoria, Australia; 11 Cancer Council Victoria, Melbourne, Victoria, Australia; 12 School of Human, Health and Social Sciences, Central Queensland University, Brisbane, Queensland, Australia

**Keywords:** Australian Aboriginal and Torres Strait Islander Peoples, Smoking Reduction, Health Equity

## Abstract

**Introduction:**

Mailed smoking and vaping cessation support provided through the *Which Way? Quit Pack* programme has demonstrated acceptability and preliminary effectiveness in delivering culturally grounded support. However, there is no evidence of the effectiveness of mailed cessation programmes for Aboriginal and Torres Strait Islander people when implemented on a national scale. Building on previous work, this study aims to evaluate both the implementation effectiveness and cost-effectiveness analysis of the *Which Way? Quit Pack* mailed smoking and vaping cessation intervention for Aboriginal and Torres Strait Islander people.

**Methods and analysis:**

A national single-group hybrid type 2 effectiveness-implementation study with 2000 Aboriginal and/or Torres Strait Islander people who use cigarettes and/or e-cigarettes (vapes) daily, want to quit and are aged ≥18 years. Participants will receive access to a culturally tailored mailed quit pack that includes up to a 12-week supply of nicotine replacement therapy, phone-based behavioural counselling and access to an online support forum. Cessation outcomes including 7-day point prevalence abstinence, prolonged abstinence and quit rates will be evaluated at 3, 6 and 12 months. An intention-to-treat approach will be used for cessation outcomes, with participants lost to follow-up classified as smokers or vapers. Exploratory logistic regression analyses will examine factors associated with quitting, and causal machine learning methods will be used to explore variation in intervention effectiveness across participant subgroups. The Reach-Effectiveness-Adoption-Implementation-Maintenance framework will guide evaluation of implementation and effectiveness outcomes. Analysis measures include cessation and quit attempt rates, number of participants recruited, representativeness and intervention uptake. The Consolidated Health Economic Evaluation Reporting Standards (CHEERS) will guide the cost-effectiveness analysis.

**Ethics and dissemination:**

This study is governed by Aboriginal and Torres Strait Islander leadership and community-controlled partnerships and is conducted in line with Indigenous data sovereignty and Indigenous research ethics principles. The study has also received approval from the following human research ethics committees: Aboriginal Health & Medical Research Council of NSW (2493/25), Aboriginal Health Committee South Australia (04-25-1209), the Western Australian Aboriginal Health (HREC1474), the Victorian Aboriginal Community Controlled Health Organisation (2025/HREC0061) and the University of Newcastle Human Research (H-2025-0311). Findings will be disseminated in forms determined, governed and endorsed by Aboriginal and Torres Strait Islander people and communities through the project Governance, including peer-reviewed publications, policy briefs, community-led knowledge translation activities and presentations at local, national and international conferences.

**Trial registration number:**

ACTRN12626000459325.

**STRENGTHS AND LIMITATIONS OF THIS STUDY**

- This hybrid type 2 effectiveness-implementation study will concurrently evaluate real-world effectiveness and implementation outcomes of an Aboriginal and Torres Strait Islander-led, governed, developed and culturally grounded intervention, designed specifically with and for Aboriginal and Torres Strait Islander peoples and communities.
- Recruitment of a large, national sample enhances generalisability of findings across diverse Aboriginal and Torres Strait Islander populations.
- The single-group study design without a comparison arm limits causal inference regarding intervention effectiveness.
- The use of self-reported cessation as the primary outcome may introduce recall and social desirability bias.

## Introduction

 Commercial tobacco and nicotine use is the most significant risk behaviour contributing to chronic disease and preventable death among Aboriginal and Torres Strait Islander people. In 2018, 12% of the total disease burden experienced by Aboriginal and Torres Strait Islander people was attributed to tobacco and nicotine use, accounting for 23% of all deaths among Aboriginal and Torres Strait Islander peoples.^1^ In 2022–2023, 20% of Aboriginal and Torres Strait Islander people smoked commercial tobacco daily.^2^ Evidence for e-cigarette use is increasing, with findings suggesting that approximately 21.6–24.6%^3 4^ of Aboriginal and Torres Strait Islander people have ever used an e-cigarette and approximately 8.6% report current e-cigarette use or vaping.^4^ Vaping is associated with nicotine-related harms including nicotine dependence, potential progression to commercial tobacco smoking and negative health outcomes including asthma, cough and injuries.^5–7^ The high prevalence of tobacco and nicotine use among Aboriginal and Torres Strait Islander people is inextricably linked to the impacts of colonisation, including the systematic introduction and embedding of commercial tobacco within Aboriginal and Torres Strait Islander communities, including as a form of payment in lieu of wages.^8^ Today, the commercial tobacco and nicotine industry continues to actively promote and market commercial tobacco, vaping and nicotine products, including actively targeting Aboriginal and Torres Strait Islander people.^9^

Reducing commercial smoking rates among Aboriginal and Torres Strait Islander people is a national priority, reflected in the National Aboriginal and Torres Strait Islander Health Plan 2021–2031^10^ and the National Tobacco Strategy.^11^ Implementing effective smoking and vaping cessation strategies is critical to achieving this goal and would significantly contribute to reducing the prevalence of chronic diseases and premature mortality, improving individual and community health, decreasing healthcare costs and reducing health disparities. While there is consistent evidence that Aboriginal and Torres Strait Islander people want to quit commercial smoking,^12–14^ only a small proportion of people report using evidence-based smoking cessation treatments that are known to increase the likelihood of quit attempts resulting in longer term cessation.^15^ The Royal Australian College of General Practitioners (RACGP) guidelines recommend the use of multisession behavioural counselling combined with pharmacotherapies, such as nicotine replacement therapy (NRT),^16^ varenicline or bupropion^17 18^ (where clinically appropriate), to support Aboriginal and Torres Strait Islander people to quit commercial smoking. However, significant structural inequities, systemic racism and other barriers persist for Aboriginal and Torres Strait Islander peoples in accessing culturally grounded and Indigenous-led behavioural and pharmacotherapy support. These include low awareness and use of available cessation supports such as Quitline,^19 20^ lack of advice and support from healthcare providers to quit^21 22^ and a lack of awareness of and trust in mainstream health systems. Together, these structural and systemic barriers highlight the urgent need for culturally grounded community-led interventions that are accessible, acceptable and responsive to the needs and priorities of Aboriginal and Torres Strait Islander peoples and communities.

In direct response to community-identified priorities, the *Which Way? Quit Pack* intervention was codesigned in 2021 in genuine partnership with peak organisations including the Aboriginal Health & Medical Research Council (AH&MRC), Quit Victoria and the Victorian Aboriginal Community Controlled Health Organisation (VACCHO).^23^ The initial *Quit Pack* intervention aimed to answer the community-led question, ‘what supports our mob to quit smoking?’, by examining the feasibility and acceptability of a mailed smoking cessation package and the uptake of a range of different behavioural and pharmacological supports. *Quit Pack* contained mailed smoking and vaping cessation support including written cessation resources, proactive referral to Quitline, mobile phone applications and the option of free combination NRT.^23^ At the time of intervention development, existing evidence suggested that interest in and uptake of NRT were low among Aboriginal and Torres Strait Islander people.^24^ However, the pilot demonstrated strong interest from Aboriginal and Torres Strait Islander people, recruiting 165 participants in 3 months (exceeding the target of 100), high uptake of NRT (>90% of participants accepted and used NRT) and achieving 7-day self-reported point prevalence smoking abstinence at 6 months of 34% (complete case) and 19.4% (intention to treat (ITT)), respectively.^25^ Building on this success and continued interest from Aboriginal and Torres Strait Islander people for mailed cessation supports, a subsequent hybrid type 1 effectiveness-implementation study was codeveloped with the established and sustained community partnerships conducted across New South Wales (NSW), Australian Capital Territory and Victoria in 2023–2024.^26^ The effectiveness-implementation trial recruited 722 participants in 9 months and achieved 7-day self-reported abstinence rates of 39% at 6-month follow-up and 44% at 3 months (complete case).

The *Quit Pack* trial used an integrated knowledge translation approach that sustained active engagement with key stakeholders including peak bodies, policy makers and Aboriginal and Torres Strait Islander communities. As the study progressed, Aboriginal and Torres Strait Islander communities in jurisdictions not originally included in the *Quit Pack* study sought involvement and, through this process, generated additional community-led research questions. In response, a formal partnership with the National Aboriginal Community Controlled Health Organisation (NACCHO) was established to provide project governance and strengthen pathways for translation of findings to policy stakeholders. This national iteration of the *Which Way? Quit Pack* study aims to support 2000 Aboriginal and Torres Strait Islander people to quit smoking and vaping, while evaluating the effectiveness at 12-month follow-up and cost-effectiveness of the intervention at a national scale. This expanded trial will provide new insights and generate evidence to inform sustainable national implementation.

### Study aims

Building on this platform of community leadership, partnership governance, iterative knowledge translation and our completed work,^25 27^ this study aims to evaluate both the implementation and effectiveness of the culturally grounded *Which Way? Quit Pack* smoking and vaping cessation intervention, developed by and for Aboriginal and Torres Strait Islander people, nationally using a single-group hybrid type 2 effectiveness-implementation study. This study will use the Reach-Effectiveness-Adoption-Implementation-Maintenance (RE-AIM) framework^28^ to evaluate the following outcomes:

1. *Effectiveness outcomes:* the primary outcome is self-reported 7-day point prevalence abstinence from smoking and/or vaping at 12-month follow-up. Secondary outcomes include: (1) self-reported 7-day point prevalence abstinence from smoking and/or vaping at 3 and 6 months; (2) prolonged abstinence, defined as no smoking or vaping from 1 month after receipt of the *Which Way? Quit Pack*, allowing for lapses of up to 7 consecutive days; (3) number of quit attempts lasting longer than 24 hours at 3 and 6-month follow-ups; and (4) biochemical verification of smoking status using expired carbon monoxide (CO) breath test at 3, 6 and 12-month follow-ups.
2. *Implementation outcomes:* implementation outcomes will include: (1) intervention reach, including participation rates and representativeness of Aboriginal and Torres Strait Islander participants across geographical areas; (2) intervention adoption, including the number and proportion of participants completing baseline and all follow-up assessments at 3, 6 and 12 months, and uptake and use of intervention components; (3) implementation, including intervention acceptability, feasibility and fidelity; and (4) maintenance, including smoking and vaping abstinence at 12-month follow-up as a proxy for sustained behaviour change, and health economic evaluation of costs and potential scalability assessed using the Short-Form 8 Health Survey (SF-8D) at baseline and 3, 6 and 12 months.

## Methods and analysis

This study is grounded in Indigenous-led and decolonising approaches to commercial tobacco and nicotine industry resistance and tobacco control. Findings from this trial will be interpreted within the context of ongoing commercial tobacco and nicotine industry activity, structural racism and regulatory environments that continue to shape smoking, vaping and nicotine-related behaviours among Aboriginal and Torres Strait Islander peoples. Observed patterns of cessation, uptake or engagement are not to be interpreted as individual success or failure, but as outcomes produced within these broader structural conditions. This interpretive positioning is intended to prevent deficit framing, avoid industry-aligned narratives of addiction as an individual or personal choice and ensure that evidence generated through this study is used to inform community-led policy and structurally responsive cessation systems, underpinned by Indigenous intellectual sovereignty and self-determination.

### Patient and public involvement

The research applies Rigney’s Indigenist research methodology^29^ and Walter and Anderson’s Indigenous quantitative methodology.^14^ The project undertook a 12-month national consultation and knowledge translation process to finalise project design, methods and data collection tools and engage Aboriginal and Torres Strait Islander people and communities across the expanded jurisdictional reach. The project team conducted community-specific meetings face to face or via Zoom, presented to and engaged with Tackling Indigenous Smoking (TIS) teams (TIS teams are Aboriginal and Torres Strait Islander-led, community-based public health teams funded by the Australian Government to reduce tobacco use and exposure to tobacco smoke. They operate nationally through Aboriginal Community Controlled Health Organisations and partner organisations, delivering culturally appropriate population-level tobacco control activities) across the country and conducted an online webinar with >150 individuals and organisations registered. The webinar introduced the team members, shared findings from previous trials and presented the research plan together with the governance and partnership model for comments. The following methods have been informed by this comprehensive Indigenous-led process.

#### Indigenous leadership and methodologies

This study is led by Aboriginal and Torres Strait Islander (MK, CC, LJW, FC) and Indigenous (RM, ALCM) researchers, community-based researchers (KR-B, ZM, TR, RK) and formal partnership with Aboriginal organisations such as NACCHO, AH&MRC and VACCHO. The research team includes non-Indigenous researchers who contribute specific expertise in behavioural science, implementation science, biostatistics, health policy and health economics to guide critical areas of research implementation. The project upholds relational, Indigenous research ethics which continues to respectfully engage and uphold the rights of Aboriginal and Torres Strait Islander people and communities to lead and govern research that is for and about them to drive real benefit and sustained impact. Community partnerships are an evolving component built on trusted relationships. All Aboriginal and Torres Strait Islander communities have the right to partner and lead this project collectively. It is anticipated that the Aboriginal and Torres Strait Islander leadership will continue to expand over the course of the project.

#### Community governance and control

This study has been codesigned with Aboriginal and Torres Strait Islander communities and will be implemented under their ongoing leadership and control. NACCHO, AH&MRC and VACCHO are key project partners who co-own the research. These partnerships build on established and sustained collaborations from the *Quit Pack* programme and, together with the Indigenous research team, form the project Governance Committee responsible for overseeing all aspects of the research including the establishment and implementation of Indigenous data sovereignty principles.^21^ The Governance Committee will meet at least three times a year throughout the project. In line with Closing the Gap Priority Reform 4,^30^ community partners have the rights to data. A data sharing application process will be established and managed by the Governance Committee through which communities can request deidentified data from their region or tailored reports addressing community questions. The project team includes trained Aboriginal and Torres Strait Islander community researchers who have co-led previous trials and will continue to co-lead this work (Roberts-Barker, Mills, Ridgeway, Kelly). The strong Aboriginal and Torres Strait Islander community partnerships, leadership and governance established over the past 3 years and two previous trials provide a strong foundation for implementing this research in accordance with Aboriginal and Torres Strait Islander community recommendations for conducting health and medical research ethically.^31^ All aspects of this work embed Aboriginal and Torres Strait Islander control and self-determination to deliver a community-driven, culturally safe, smoking and vaping cessation study as evidenced in our early phases.^12 19 20^

### Study design and implementation framework

This study will use a pragmatic hybrid type 2 effectiveness-implementation design^32^ to examine the impact of the *Which Way? Quit Pack* intervention on individual cessation outcomes, while also gathering data to evaluate the implementation and inform sustainable national implementation. This design was chosen in recognition of the challenges of implementing high-quality controlled trials in Indigenous context broadly,^33^ and specifically in Indigenous smoking cessation studies,^34^ and to provide all participants who want to quit with immediate support. Evaluation of study implementation outcomes will be guided by the RE-AIM framework^28^ to assess programme outcomes comprehensively to inform and enhance real-world integration.

### Study setting

The national study will be implemented in all states and territories of Australia.

### Participant eligibility

Individuals will be eligible to participate if they self-identify as Aboriginal and/or Torres Strait Islander, use cigarettes and/or e-cigarettes (vapes) daily, want to quit and are aged ≥18 years. Pregnant participants will not be excluded from the study. This is consistent with current RACGP guidelines, which state that NRT may be recommended for pregnant women who are unable to quit smoking with behavioural support alone.^35^ Participants who are pregnant at enrolment or become pregnant during the trial will be advised to seek advice from their general practitioner before using NRT. Individuals will be deemed ineligible if they are currently enrolled in any other smoking or vaping cessation programme, self-report a previous allergy or hypersensitivity to nicotine or any ingredient in NRT products, or report experiencing any health condition in the 6 weeks prior to enrolment that is a contraindication for NRT use. This includes heart attack, unstable angina, irregular heart rhythm, stroke/ministroke, brain aneurysm or vascular malformations.

### Participant recruitment

Recruitment approaches have been informed by previous *Which Way? Quit Pack* pilot studies, which recruited 50–200 participants per month.^25^ Participants will be recruited via self-referral using three main strategies: direct text messaging, social media promotion and dissemination of printed materials and community engagement. Each strategy will direct people to the study landing page in REDCap, where participants can access detailed information, review eligibility criteria and complete the online consent (see online supplemental file 1) and registration process.

1. *Direct text message outreach*. A database of >700 individuals who have previously expressed interest in participating in *Which Way? Quit Pack* will be sent a direct text message by the research team via a third-party text platform to inform them that recruitment for the current study is open and direct them to the study landing page. Individuals in the database include participants from previous trials who did not quit, social media followers, webinar attendees and community members who have requested notification about future studies.
2. *Social media recruitment*. A targeted digital campaign will be implemented across social media platforms. The campaign will include: (1) paid, targeted advertisements using Meta for Business (Facebook and Instagram posts); (2) organic posts shared via *Which Way?* social media accounts; and (3) amplification of content through partnership organisations, community-controlled health service and Aboriginal and Torres Strait Islander community networks sharing study promotional materials. Social media advertisements will include a URL and/or QR code to direct participants to the study landing page.
3. *Printed materials and community engagement*. Printed promotional materials advertising the study will be distributed through partnering Aboriginal and Torres Strait Islander communities, Aboriginal Community Controlled Health Services and at relevant community events. This will include promotion of the study at large national community events, including relevant conferences and workshops. All printed materials will include a URL and/or QR code to direct participants to the study landing page.

### *Quit Pack* intervention

**Figure 1:**
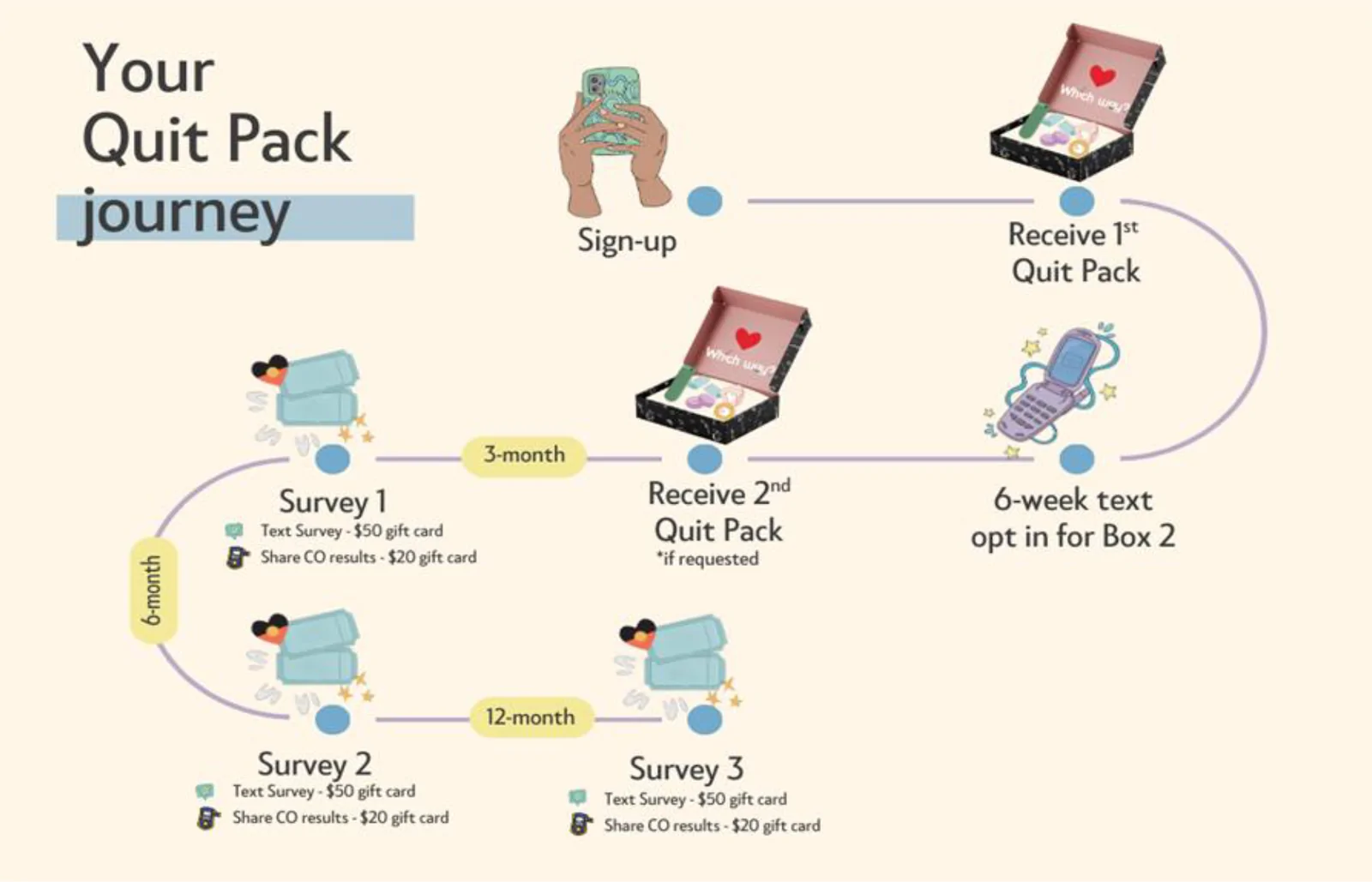
Overview of participant enrolment, *Quit Pack* delivery and follow-up assessments. CO, carbon monoxide.

The *Which Way? Quit Pack* intervention comprises three core components designed to support Aboriginal and Torres Strait Islander peoples in their quit journeys by providing culturally grounded and evidence-based care. An overview of participant processes for receiving quit packs and completing data collection is outlined in figure 1.

#### Mailed quit pack, inclusive of NRT

**Figure 2:**
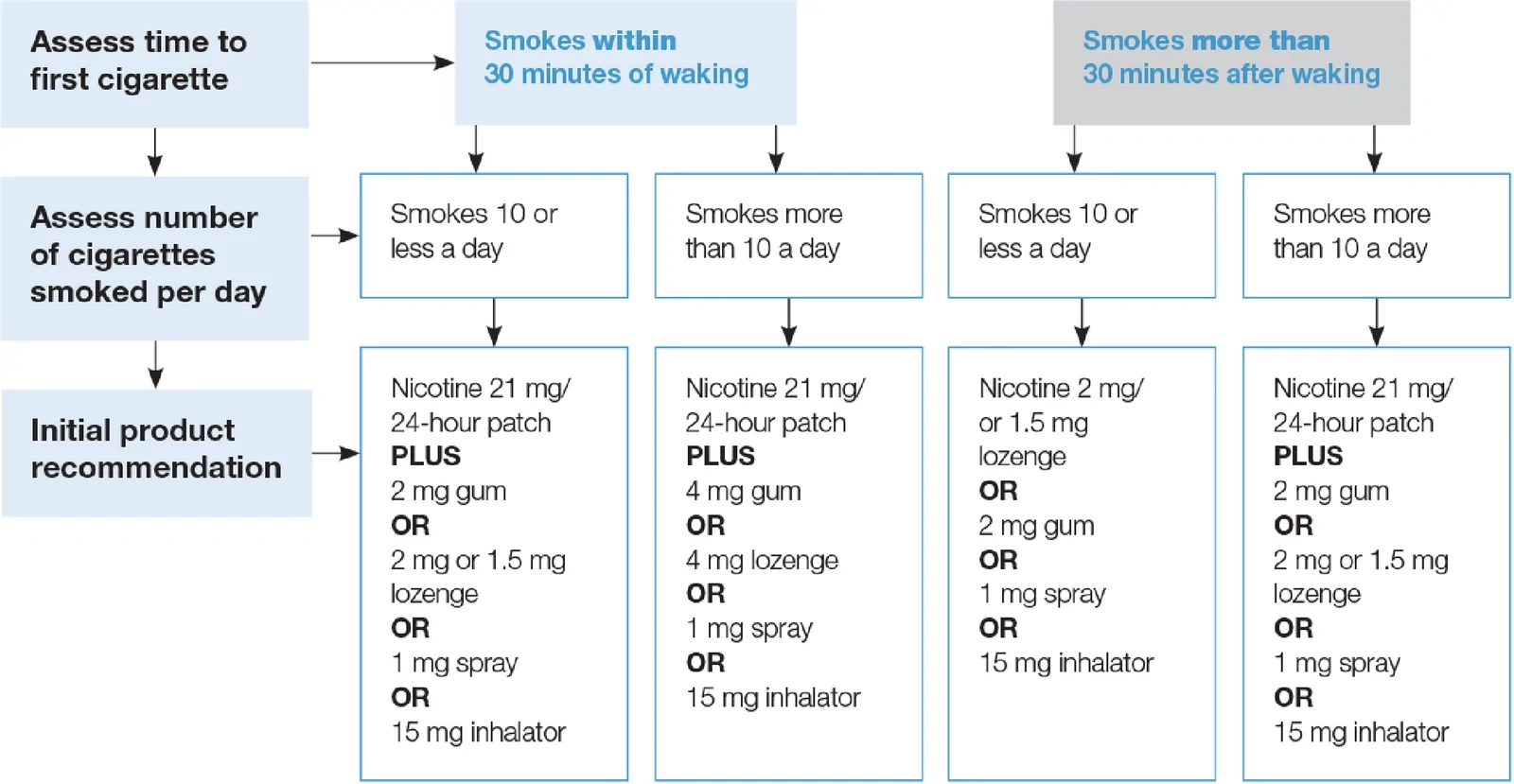
Royal Australian College of General Practitioners (RACGP) dosage guidelines.

Participants who enrol in *Which Way? Quit Pack* will receive a comprehensive mailed smoking cessation intervention delivered to their nominated postal address. Each *Quit Pack* will include: (1) merchandise from Aboriginal-led, majority Aboriginal-owned accredited social enterprise ‘Clothing the Gaps’ to celebrate pride in culture; (2) a written health education story book developed specifically by the Indigenous *Which Way?* team that offers culturally relevant advice and guidance on quitting and NRT use; (3) an iCOquit Smokerlyzer (for those who report using tobacco only), which is a handheld device that measures CO levels in exhaled breath, supporting self-monitoring and motivation; and (4) a maximum 12-week supply of NRT (see table 1). The *Quit Pack* box and its presentation also embed culturally relevant, engaging and visually meaningful elements throughout, creating an experience that reflects and celebrates Aboriginal and Torres Strait Islander cultures and supports participants to feel culturally represented and safe when receiving and engaging with the intervention. Participants will receive NRT in two separate mailouts. The first will be sent at baseline and will include an 8-week supply of NRT. Six weeks post-enrolment into the intervention, participants will receive an SMS or email (as per their selected preference) asking them to confirm if they wish to receive the further 4-week supply. On confirmation, the second shipment will be mailed (see figure 2). NRT is available over the counter in Australia and does not require a prescription for use.

**Table 1:** NRT options for participants to select at baseline (8-week supply) and follow-up (4-week supply)

		NRT types supplied	High-dose pack	Low-dose pack
Baseline supply (8-week supply)	Option 1—cNRT	Patch, gum and lozenge	2 boxes of 25 mg patches* 4 boxes of 4 mg gum 4 packs of 4 mg lozenges	2 boxes of 25 mg patches* 4 boxes of 2 mg gum 4 packs of 2 mg lozenges
	Option 2—cNRT	Patch, mouth spray and gum	2 boxes of 25 mg patches 4 packs of mouth spray 4 boxes of 4 mg gum	2 boxes of 25 mg patches* 4 packs of mouth spray 4 boxes of 2 mg gum
	Option 3—cNRT	Patch, mouth spray and lozenge	2 boxes of 25 mg patches 4 packs of mouth spray 4 packs of 4 mg lozenges	2 boxes of 25 mg patches* 4 packs of mouth spray 4 packs of 2 mg lozenges
	Option 4—oral NRT only	Gum, mouth spray and lozenge	3 boxes of 4 mg gum 2 packs of mouth spray 3 packs of 4 mg lozenges	3 boxes of 2 mg gum 2 packs of mouth spray 3 packs of 2 mg lozenges
Follow-up supply (4-week supply)	Patches		1 box of 25 mg patches	4 boxes of 15 mg patches
	AND/OR		AND/OR	AND/OR
	Oral NRT (Participants select up to 4 packs in total)		A selection of the following (up to 4 packs in total): 1 pack of 4 mg lozenges 1 pack of 4 mg gum 1 pack of mouth spray	A selection of the following (up to 4 packs in total): 1 pack of 2 mg lozenges 1 pack of 2 mg gum 1 pack of mouth spray

Box quantities/pieces per box: 25 mg patches—1 box=28 patches; 15 mg patches—1 box=7 patches; gum (4 and 2 mg)—1 box=75 pieces; lozenges (4 and 2 mg)—1 pack=80 lozenges; mouth spray—1 pack=2 mouth sprays.

* Participants will be able to exchange the 2 boxes of 25 mg patches for 8 boxes of 15 mg patches if a lower dose is preferred.

cNRT, combination NRT; NRT, nicotine replacement therapy.

Participants will have the opportunity to select one *Quit Pack* NRT box (see table 1) based on their NRT preferences, including combination NRT options, oral NRT only or patch only (for the second mailing only). The staged delivery of NRT is designed to balance clinical effectiveness, participant autonomy and efficient resource use. Provision of a standardised 8-week baseline supply supports early quit attempts by reducing decision burden at enrolment and ensuring access to evidence-based dosing during the period of highest relapse risk. Each participant will receive a high-dose or low-dose pack, based on their self-reported baseline smoking and vaping behaviours (see figure 1). The follow-up 4-week supply allows participants to select their NRT based on lived experience of cravings, product acceptability and adherence and will be selected by participants during confirmation of additional supply. Dosage of NRT is in line with RACGP guidelines for smoking cessation. In the absence of specific evidence about dosage of NRT for vaping, the same guidelines will be used to support vaping cessation.^36^ Nicotine mouth spray was approved by the Therapeutic Goods Administration in October 2025 for treatment of vaping dependency^36^ and will be included as an NRT option. Participants will have the opportunity to titrate their patch dosage and request 15 mg patches if preferred. *Quit Pack* NRT configurations are informed by data on the most requested NRT types from previous trials,^25^ including the consideration of allowing participants to select lower dosage patches based on their personal preference. As oral NRT is the recommended first-line treatment during pregnancy, participants who are pregnant at enrolment will receive a *Quit Pack* containing oral NRT only. Participants who report low weight (<45 kg) will also only be offered an oral NRT *Quit Pack* box.

Participants will receive an information booklet in their packs which provides: an introduction to the Aboriginal and Torres Strait Islander *Which Way?* team members, including photos, details on how to use the CO monitor (if included in their pack), how to access phone support, how to access and register for the online forum, how to contact the team and a Quit Plan, and an overview of the research process (ie, follow-up time points). The sharing of CO monitor data has been described in a video developed by the *Which Way?* team. The developed *Quit Pack* resources have been designed by and for Aboriginal and Torres Strait Islander peoples and communities, embedding Aboriginal and Torres Strait Islander artwork, ways of knowing, being and doing as central to communicating and translating a range of Indigenous-led educational content and supports. This culturally grounded approach ensures the relevance and responsiveness of all aspects of *Quit Pack* to Aboriginal and Torres Strait Islander peoples and communities, from conception through implementation and beyond.

#### Phone-based behavioural counselling with the *Which Way?* cessation support team

Participants will have access to unlimited phone-based behavioural counselling during business hours (Monday–Friday, 9:00 am–5:00 pm) delivered by one of four Aboriginal and Torres Strait Islander team members (men and women). Team members have completed training through the Nicotine Addiction, Smoking and Vaping Cessation course provided by Avondale University. The *Which Way?* counselling model builds on insights from two previous *Which Way?* trials^25^ and is designed to provide culturally grounded, participant-led support. Participants can opt in to receive behavioural counselling via an information card included in their *Quit Pack,* which will direct them to an online booking system. The cessation support team will deliver tailored guidance on behavioural strategies for smoking and/or vaping cessation and NRT use,^37^ guided by best evidence practice and individual participant needs. The cessation support team will receive ongoing clinical supervision from an Aboriginal medical doctor, with workload management strategies in place to ensure staff well-being and high-quality care.

#### Access to online information and support

All participants will have access to online information and support to assist their quit attempt. Information content will be hosted on the *Which Way?* website and will include educational content and downloadable resources developed specifically by and for Aboriginal and Torres Strait Islander people to support their smoking and vaping cessation journeys. These resources have been codesigned as part of previous trials and include interactive tools to assist with goal setting, tracking progress and staying motivated. Participants will also have access to a secure, password-protected online forum hosted through the existing *Which Way?* website and available as a free downloadable mobile app (available for Apple iOS and Android). The forum provides a space for participants to share experiences and quit stories and connect with others in the programme. The forum will also be used to share health education content, links and videos relevant to smoking and vaping cessation. The forum will be managed by the *Which Way?* team who have received training and education in smoking and vaping cessation and hold or are studying towards tertiary qualifications in social work, medicine and psychology.

### Data collection and measures

Data will be collected between 29 May 2026 and 31 May 2030. Outcome measures and data collection tools have been developed through a series of collaborative workshops with our internal project team, Indigenous investigators and community partners, who have approved our final data collection tools. “It is the powerful influence of the usually invisible standpoints that inform what data are gathered, by whom, and for what purpose” (p 56). In upholding Indigenous quantitative methodologies, the *Which Way?* project acknowledges *“*the extent to which the very categories used to collect statistical information profoundly shape the kinds of interpretations possible” (p 27)*.*^38^

### Outcomes

#### Effectiveness outcomes

*Seven-day point prevalence abstinence*. The primary effectiveness outcome is 7-day point prevalence abstinence, which will be measured at 3, 6 and 12-month follow-ups. This outcome was selected because it minimises recall bias by capturing recent smoking and/or vaping behaviour over a short and clearly defined timeframe. It also aligns with the structure of the intervention, as participants are encouraged, but not required, to set a quit date, and the timing of quit attempts is expected to vary across participants making point prevalence abstinence the most appropriate and comparable outcome. Self-reported 7-day abstinence rates will be assessed in line with recommendations from the Society for Research in Nicotine and Tobacco but modified for the study population.^27^ Seven-day point prevalence abstinence will be derived as answering ‘yes’ to the question, “In the last 7 days, have you stayed smoke and/or vape free (not even a puff)?”. ‘Vape’ will replace ‘smoke’ to assess vaping cessation. Dual cessation will be defined as answering ‘yes’ to both questions.

*Prolonged abstinence* will be measured at 3, 6 and 12 months and defined as continuous abstinence from smoking or vaping from 1 month after receipt of the *Which Way? Quit Pack*, allowing for lapses of up to 7 consecutive days, consistent with established smoking cessation outcome definitions.^39^ Assessment of prolonged abstinence will be restricted to participants who report **7-**day point prevalence abstinence at each follow-up. Participants will be asked, “At any time from 4 weeks after receiving your quit pack, have you smoked for 7 days in a row?”*.* Those who answer ‘no’ will be defined as having achieved prolonged abstinence. Those who answer ‘yes’ will be defined as not achieving prolonged abstinence (had a relapse). Participants who report relapse at any assessment will not be classified as prolonged abstinent at subsequent time points.

*Quit attempts* will be assessed at 3, 6 and 12-month follow-ups. A quit attempt will be defined as a period of intentional abstinence from smoking or vaping lasting longer than 24 hours. At each follow-up time point, quit attempts will be assessed among participants who report ongoing smoking or vaping, or who have relapsed since the previous survey. Participants who report continuous abstinence since the last assessment will not be asked to report quit attempts for that period. At 3 and 6-month follow-ups, participants will be asked, “In the last three months, how many times have you made a quit attempt lasting longer than 24 hours?”, and requested to enter the number of times they have made a quit smoking attempt. At 12-month follow-up, participants will be asked, “In the last six months, how many times have you made a quit attempt lasting longer than 24 hours?”, and requested to enter the number of times they have made a quit smoking attempt.

*Biochemical verification of smoking status*. All participants who reported at baseline who want to quit smoking cigarettes will be invited to undertake biochemical verification of smoking status at 3, 6 and 12-month follow-ups. Verification will be optional and will be conducted using CO breath test via an iCOquit Smokerlyzer, which is provided to participants as part of their *Quit Pack*. The Smokerlyzer connects to an app on the participant’s phone/tablet, enabling participants to self-administer the test. Participants will be asked to submit their CO reading to the research team via text message. This approach enables objective verification of recent smoking status while minimising participant burden and maintaining the remote, community-based nature of the intervention. A cut-point of 6 parts per million will be used to define non-smoking, in line with Society for Research in Nicotine and Tobacco guidelines.^40^ Participants will receive a $20 gift card as reimbursement for the time and effort required to complete each biochemical verification. As biochemical verification of smoking status has not previously been piloted in this intervention setting, data generated through this process will also inform adoption and implementation outcomes, including feasibility, acceptability and participant adherence to verification procedures. These findings will support refinement of verification processes for use in future trials with Aboriginal and Torres Strait Islander people.

#### Implementation outcomes

Implementation outcomes will be assessed across five domains (see table 2). Reach will be measured by the number, proportion and representativeness of Aboriginal and Torres Strait Islander participants, including demographic characteristics and current access and use of Aboriginal health services, obtained from baseline participant surveys. Adoption will be measured by the number, proportion and representativeness of participants who complete all baseline and follow-up assessments and uptake of intervention components, including *Quit Pack* resources and NRT, phone calls with *Which Way?* team members and participation in the online forum. These data will be obtained from trial tracking systems, including data kept by the research team on NRT pack requests, behavioural supports provided and the number of posts on the online forum. Implementation will be measured by assessing contextual adaptations made to the intervention recorded via the community researchers’ project implementation logs and weekly project meetings. Weekly project meetings will follow a structured format guided by three key questions: (1) what worked well, (2) what has not worked well and (3) what changes are needed to improve delivery. These discussions will focus on fidelity to intervention protocols, quality and consistency of delivery and contextual factors influencing implementation. For this trial, maintenance outcomes have been conceptualised to include individual-level cessation outcomes, reflecting the importance of sustained abstinence and ongoing quit attempts as key indicators of long-term intervention impact in smoking and vaping cessation.^41^ Maintenance will therefore be determined by prolonged cessation and quit attempts at 12-month follow-up, as well as an assessment of the cost-effectiveness of the intervention as an indicator of programme-level sustainability, as found appropriate in a previous work.^27^

**Table 2:** Summary of effectiveness and implementation outcome measures

Dimension	Outcome	Data collection source Analysis plan	Data collection time points
**Reach** Determine the extent to which the *Which Way? Quit Pack* intervention reaches the target population of Aboriginal and Torres Strait Islander people who smoke daily and/or vape daily*.*	Number of participants enrolled in the trial.	Participant survey Quantitative analysis	Baseline
	Participant representativeness including age, gender, geographical location, remoteness classification (using the Socio-Economic Indexes for Areas (SEIFA)) and current access and use of Aboriginal health services.	Participant survey Quantitative analysis	Baseline
**Effectiveness** Determine the impact of the *Which Way? Quit Pack* intervention on smoking and vaping cessation rates at 3, 6 and 12-month follow-ups, with a primary focus on abstinence at 12 months.	Self-reported 7-day prevalence abstinence.	Participant survey Quantitative analysis	3, 6 and 12 months post-baseline
	Prolonged abstinence.	Participant survey Quantitative analysis	3, 6 and 12 months post-baseline
	Number of quit attempts lasting ≥24 hours.	Participant survey Quantitative analysis	3, 6 and 12 months post-baseline
	Biochemical verification of smoking status.	Carbon monoxide via Smokerlyzer Quantitative analysis	3, 6 and 12 months post-baseline
**Adoption** Determine the extent to which participants use each component of the *Which Way? Quit Pack* intervention*.*	Absolute number and proportion of participants who complete each survey.	Participant survey Quantitative analysis	Baseline and 3, 6 and 12 months post-baseline
	Representativeness (age, gender, geographical location, remoteness classification (using SEIFA)) of participants who complete each survey.	Participant survey Quantitative analysis	Baseline and 3, 6 and 12 months post-baseline
	Uptake and frequency of use of intervention components including nicotine replacement therapy (NRT), phone-based behavioural counselling and participation in the online forum.	Data from trial tracking systems (NRT pack requests, behavioural support records and online forum use) Quantitative analysis	Baseline to end of intervention (3-month follow-up)
**Implementation** Determine the fidelity, quality and consistency of the *Which Way? Quit Pack* intervention delivery, as well as the barriers and facilitators encountered during the implementation process*.*	Qualitative process evaluation using staff feedback to assess fidelity, barriers and facilitators to delivery. Evaluation will be guided by three questions: (1) ‘what worked well?’ to identify effective strategies, resources or processes supporting implementation; (2) ‘what has not worked well?’ to explore challenges, barriers or gaps experienced; and (3) ‘what changes need to be made?’ to gather suggestions for addressing barriers and improving implementation.	Implementation review meetings with project staff Qualitative analysis	Weekly
**Maintenance** Determine the long-term sustainability of the *Quit Pack* intervention on smoking and vaping cessation at 12-month follow-up and conduct a health economic evaluation to inform scalability and sustainability of the intervention for continued use and support within the community and healthcare settings beyond the study period.	Self-reported 7-day point prevalence abstinence at 12-month follow-up.	Participant survey Quantitative analysis	12 months post-baseline
	Prolonged abstinence at 12-month follow-up.	Participant survey Quantitative analysis	3, 6 and 12 months post-baseline
	Optional biochemical verification of smoking status at 12-month follow-up.	Participant survey Quantitative analysis	12 months post-baseline
	Cost-effectiveness of the intervention including cost per participant, health-adjusted life years gained, hospitalisations averted and number of quitters.	Participant surveys using Short-Form 8 Health Survey (SF-8D) Quantitative analysis	Baseline and 3, 6 and 12 months post-baseline

### Sample size

A sample size of 2000 eligible consenting participants will enable the study to estimate the primary effectiveness outcome proportions and 95% CIs with a margin of error between 0.8% and 2% for proportions that range between 5% and 50% (and by symmetry as high as 95%). Assuming a minimum 12-month cessation 7-day point prevalence of 5%, this sample size will give the study over 80% power to detect differences in the secondary outcomes. We will use a type 1 error rate of 5%. Pilot data indicated potential 7-day point prevalence at 3 and 6 months of 23% and 16%.^25^

### Statistical analysis

The representativeness of the sample will be assessed by comparing observed proportions to expected proportions derived from Australian Bureau of Statistics population data using χ^2^ goodness-of-fit tests. The RE-AIM quantitative components will be described as counts and percentages with 95% CIs, and qualitative components will be thematically analysed (see table 2). Associations between participant demographic characteristics and quit rates will also be estimated using logistic regression models, as secondary exploratory analyses. Causal machine learning models^31^ will be used to assess potential heterogeneity in effectiveness of implementation strategies by participant subgroups. An ITT analysis will be performed for all effectiveness cessation outcomes, where participants with missing smoking or vaping cessation outcomes are classified as smokers or vapers. Multiple imputation using chained equations will be used to classify all remaining missing outcomes, as well as missing data on confounders. Attrition will be reported in the participant flow chart, and the baseline demographic and smoking/vaping characteristics of completers and non-completers will be compared via t-tests and χ^2^ tests as appropriate. Complete case results will also be reported to provide transparency and allow comparison across analytical methods.^42^ The proportion of participants with a quit attempt longer than 24 hours will be estimated within the subgroup of participants who have not been continuously abstinent at the 3, 6 and 12-month follow-ups.

### Cost-effectiveness analysis

The cost-effectiveness analysis (CEA) will follow the Consolidated Health Economic Evaluation Reporting Standards (CHEERS 2022), assessing both costs and health outcomes associated with national delivery of the *Which Way? Quit Pack*. A trial-based CEA will be conducted from the health system perspective, capturing all intervention delivery costs (NRT supply, postage, staffing, behavioural counselling, digital infrastructure and administrative overheads) and participant healthcare utilisation. Resource use will be tracked prospectively using project financial records and participant-reported healthcare contacts, with unit costs sourced from standard Australian cost schedules. Health-related quality of life will be measured using the SF-8D, enabling estimation of quality-adjusted life years (QALYs). Incremental cost-effectiveness ratios will compare intervention costs with QALYs gained and with validated cessation outcomes. Sensitivity analyses will test assumptions around missing data, NRT uptake and cost variation.

## Discussion

Commercial tobacco use among Aboriginal and Torres Strait Islander peoples must be understood within the ongoing impacts of colonisation, racism and commercial/industry exploitation. Commercial tobacco was systematically introduced and normalised through colonial systems, including as payment in lieu of wages, and its harms have been sustained through targeted marketing, regulatory neglect and the prioritisation of industry interests over health and sovereignty. Therefore, contemporary commercial tobacco use and vaping patterns reflect structural conditions rather than individual choice or failure, underscoring the need for responses that are Indigenous led, culturally grounded and structurally informed. This large-scale trial will be the first to test the effectiveness of a mailed cessation intervention for Aboriginal and Torres Strait Islander people on a national scale.

This study has a number of strengths, including its pragmatic, real-world design, anticipated recruitment of a large national sample and the use of a comprehensive evaluation framework that includes examination of both effectiveness and implementation outcomes. To our knowledge, *Quit Pack* is the first national Aboriginal and Torres Strait Islander smoking and vaping cessation intervention to embed Indigenous leadership and governance throughout all aspects of intervention development, design and delivery, reflecting a culturally grounded and self-determined approach. However, some limitations should be noted. The single-group design without a comparison arm limits the ability to attribute observed effects solely to the intervention. As such, cessation rates will be interpreted as estimates of real-world outcomes. Reliance on self-reported cessation outcomes may introduce recall and social desirability bias and influence estimated abstinence rates, although this is partially mitigated through optional biochemical verification, which will also provide critical feasibility data for implementation into future studies. Finally, while the study is designed to maximise reach and feasibility of delivering cessation support to a large and diverse group of Aboriginal and Torres Strait Islander people, the pragmatic considerations for a trial of this nature necessarily limit the degree of experimental control, including obtaining detailed data about intervention fidelity, adherence and the relative contribution of individual intervention components, which may impact the generalisability of findings.

### Ethics and dissemination

This project will be conducted in line with Aboriginal and Torres Strait Islander health and medical research ethics guidelines^43 44^ and practices identified as a priority for Aboriginal and Torres Strait Islander communities.^31^ In line with these frameworks, we implement ethics as an ongoing, relational process that extends beyond formal approvals. Our approach upholds community-led, place-based rights, governance and decision-making, recognising Aboriginal and Torres Strait Islander peoples as custodians of their own knowledges and data. The *Which Way? Quit Pack* was developed in 2021 in direct response to priorities identified by Aboriginal and Torres Strait Islander communities. Since its inception, *Quit Pack* has continued to evolve through genuine, long-term partnerships with communities and organisations at local, state and national levels. The development of this current iteration has been shaped by extensive consultation, ensuring the project remains grounded in community benefit, reciprocity and relational accountability. Peak community-controlled organisations in Aboriginal and Torres Strait Islander health—including the AH&MRC and VACCHO, who have been partners since the earliest stages of *Quit Pack—*have played a central role in guiding and affirming the project’s alignment with community ethical principles and best practice. Their leadership has ensured the relevance and integrity of the project, and that it remains aligned and accountable to upholding ethical practices as defined and prioritised by Aboriginal and Torres Strait Islander communities. Ethics approval has been obtained from the AH&MRC Human Research Ethics Committee of NSW (2493/25), the Aboriginal Health Committee South Australia Ethics Committee (04-25-1209), the Western Australian Aboriginal Health Ethics Committee (HREC1474), the VACCHO (2025/HREC0061) and the University of Newcastle Human Research Ethics Committee (H-2025-0311) and is registered with ANZCTR (ACTRN12626000459325). Informed written consent will be obtained from all participants included in this study. Participant information will be kept confidential, and no data will be published that identify individual participants. Data will be collected, stored and used in accordance with the National Health and Medical Research Council data management policy.^45^

Study findings will be disseminated at local, national and international conferences and through pathways determined, governed and endorsed by the project Governance and Aboriginal and Torres Strait Islander peoples and communities. Findings will also be disseminated via peer-reviewed publications in relevant journals and policy-relevant briefs. A range of community-led knowledge translation activities and resources will be developed and shared in response to the priorities, needs and interests of participants and community partners, including localised community reports, with all translation activities designed to generate reciprocal benefit and remain accountable to community-identified priorities.^46^ These activities will include, but will not be limited to, infographics, webinars, short videos and brief reports. Knowledge translation will occur throughout the life of the study and beyond using an integrated knowledge translation approach to ensure that dissemination is not just treated as an endpoint but as an ongoing, collaborative and relational process that centres Aboriginal and Torres Strait Islander peoples and communities as the primary and enduring beneficiaries of this work.

## Supplementary material

10.1136/bmjopen-2026-121440 online supplemental file 1
