# Supplementary material for: Which Way? Quit Pack study protocol: a national hybrid type 2 effectiveness-implementation study of mailed smoking and vaping cessation support for Aboriginal and Torres Strait Islander people
Source: BMJ Open. 2026 Sep 11;16(9):e121440. doi: 10.1136/bmjopen-2026-121440 (PMC13583795; doi:10.1136/bmjopen-2026-121440)
Supplement: Supplementary file 1 [file bmjopen-2026-121440.reviewer_comments.pdf]

PEER REVIEW HISTORY

BMJ Open publishes all reviews undertaken for accepted manuscripts. Reviewers are asked to complete a checklist review form and are provided with free text boxes to elaborate on their assessment. These free text comments are reproduced below.

ARTICLE DETAILS

Title (Provisional)

Which Way? Quit Pack Study Protocol: A national hybrid type 2 effectiveness-implementation study of mailed smoking and vaping cessation support for Aboriginal and Torres Strait Islander people

Authors

Booth, Kade; Bryant, Jamie; Roberts-Barker, Kayden; Mills, Zabowie; Ridgeway, Tanika; Kelly, Raymond; Collis, Felicity; Bonevski, Billie; Maddox, Raglan; Whop, Lisa J; Chamberlain, Catherine; Martiniuk, Alexandra LC; Belfrage, Mary; Segan, Cathy; Doran, Christopher; Oldmeadow, Christopher; Leigh, Lucy; Kennedy, Michelle

---

VERSION 1 - REVIEW

**Reviewer**	**1**
**Name**	**Oesterle, Sabrina**
**Affiliation**	**Arizona State University, School of Social Work**
**Date**	**04-Jun-2026**
**COI**	**None**

I would like to disclose that I involved a graduate student, Zoe Reep in the review of this manuscript.

Affiliation: School of Social Work at Arizona State University

COI: None

This manuscript is a protocol for a hybrid effectiveness-implementation trial for a smoking and vaping cessation program targeting Aboriginal and Torres Strait Islander people, the *Which Way? Quit Pack* intervention. The trial is a collaboration between a few Aboriginal health organizations, designed following a pilot of the program and a successful Type I effectiveness-implementation trial. The protocol is registered with ANZCTR. There are several strengths to this study. First, the authors have worked extensively with the Aboriginal and Torres Strait Islander communities to design this study through community meetings, a webinar, partnerships with Aboriginal organizations, training community researchers, guidance from Aboriginal and Torres Strait Islander ethics guidelines, and ethics approval from four Aboriginal health committees. In addition, the study follows a successful pilot and Type I effectiveness/implementation study. The researchers have clearly considered the

health factors of their potential sample, including the frequency, timing of smoking, pregnancy status, and weight, and designed a variety of cessation packets to meet the needs of their target population. Finally, the authors take a clear decolonial and community-based approach, describing in-depth power, confidentiality, and dissemination. Despite these strengths, there are significant gaps in this study that may compromise the findings. Most prominent is the single-group study design, specifically the lack of a comparison group, which severely limits the evaluation of the effectiveness of the intervention. In addition, the analysis plan is questionable and entirely missing for the implementation aspect of the study. Finally, the measures for the study are not substantiated and do not meet the paper objectives. While this review follows the outline of the manuscript, these three gaps are the most important to address. Each review section also includes minor issues in the manuscript that need to be addressed.

The objectives of this study are to evaluate the effectiveness and implementation of the smoking and vaping intervention. Given prior evidence for the effectiveness of the intervention, stronger justification for why more effectiveness data are needed, specifically a Type II study. Overall, while the objective evaluating effectiveness is described in detail throughout the manuscript (measures and analyses), the objective evaluating implementation is underdeveloped, a concern considering the objective is half of the study.

The single-group study design limits rigor and the ability to answer the research question. A single-group design can show change over time, but not causal effectiveness. The design is especially limited if the study is meant to assess both effectiveness and implementation, because implementation success can be mistaken for intervention effectiveness when there is no comparison condition. The single group design is a reasonable pilot or early-stage design, but not a strong evaluation design. A community- or service-level stepped-wedge cluster randomized trial with embedded implementation evaluation would be a stronger choice. Alternatively, the authors may consider a wait-list randomized design, which can address ethical concerns around providing all participants who want to quit with support but is less useful if contamination is likely. Or consider a cluster randomized rollout by service or community. The balance between scientific rigor and ethical considerations needs to be more carefully considered and justified regardless of study design, but especially if the authors choose to retain the single-group study design. For instance, the authors indicate the study design was chosen "in recognition of the challenges of implementing high quality controlled-trials in Indigenous context" (p. 10). Rephrasing this sentence to include a few of the challenges that are being addressed through this methodology would better explain the connection.

The measures for the study need more explanation. I agree that intervention reach, including participation rates and representativeness, and intervention adoption measured as uptake and use of intervention components are intervention outcome measures. However, the number and proportion of participants completing baseline and follow-up surveys is not an implementation outcome measure (though important to track to assess and describe research study and data quality). Similarly, the proposed measure of implementation maintenance (i.e., smoking and vaping abstinence at 12-month follow-up) is not a measure of implementation sustainment but effectiveness sustainment. I recommend considering a different measure that assesses continued implementation of the intervention (e.g., the proportion of clinics/services still delivering the intervention with acceptable fidelity after active implementation support ends). In RE-AIM, maintenance is defined at the setting level as whether a program becomes part of routine organizational practice, not sustained clinical

effectiveness.

I also have questions about participant-reported behaviors. Participants will be asked to provide the number of times they have made a quit smoking attempt lasting longer than 24 hours in the last six months. Will participants be able to answer this accurately? Do the authors have information from prior work that this works? Since biomedical verification is voluntary and there is no comparison group, this measure is likely to be biased due to self-selection. I suspect that people who quit will be more likely to agree to biomedical verification than those who relapsed. Although biochemical validation is a strength of the study, the study design will likely not provide useful data. Could you design a small randomized sub-study instead to test this, especially since the biochemical verification of smoking status has not previously been piloted in this intervention setting?

Most of the measures section focuses on the first aim, leaving many unanswered questions related to the second aim. For instance, the attention to intervention cost in the setup of the study is a strength, but the assessment and analysis of cost effectiveness are not well described. What kind of cost data will be collected and how? How will this be analyzed? Why is there a focus on cost effectiveness versus other cost analysis? This is not well justified or described. Finally, the authors propose to measure implementation fidelity, quality, and consistency only with qualitative measures. There seems to be a missed opportunity for quantitative measurement as well.

Some aspects of the intervention could be explained in greater detail. Participants will receive a comprehensive information booklet, which includes details on the additional supports on offer. What are those additional supports? The authors claim the intervention provides culturally grounded support, but do not explain what that means in the context of this specific study and population. Please explain and maybe provide a few examples.

There are some gaps in the sample recruitment, treatment assignment, and data collection procedures that would offer greater insight into the study. First, more details about social media recruitment would be useful. For example, what are hierarchical advertising structures? How do algorithms target, that is, based on what? What are organic posts? Where are they placed, on what social media platforms? Also, three participant recruitment strategies are proposed, informed by previous pilot studies. Given that data from prior work is available, please provide justification for the use of all three, drawing on previous studies and literature.

Second, in the sample size justification, a range of 5% to 50% is used. That is a very big range. This needs to be more clearly justified as a useful range. What are expected cessation numbers?

Third, how will medical conditions be confirmed to recommend the right NRT? Are a doctor's evaluation and prescription needed? If not, why not? The NRT options seem complex. Are the participants selecting a treatment option, or the researchers? If the participants choose, how will people decide what is best for them? Especially between the high dose and low dose options. Will the study provide a recommendation?

Finally, the data collection procedure for the implementation aim of the study is absent. For instance, the qualitative process evaluation using staff feedback is described nowhere in the protocol text, leaving questions about the sample and recruitment (Who counts as project staff? How are/will they be trained?), data collection procedures (How will/what data be documented? What questions will be asked, if any? What is the frequency and duration of the review meetings?), analysis plans (How will fidelity, quality, and consistency be evaluated with a qualitative approach?), and ethical considerations

unaddressed.

The analysis section is poorly organized, described, justified, and linked to the objectives of the study. What is the primary analysis for the effectiveness question? And what are the primary statistical analyses/models? Will there be pre-post analyses? Since there is no comparison group, how will we know that effects are really due to the intervention, and which component? Since there are so many components, they need to be explored - how do we know it's NRT or counseling etc.?

No rationale is given for the inverse probability of treatment weighting; please provide, along with a definition of “intention to treat.” Since subgroup analyses are proposed, the power analyses need to include power estimates for these secondary analyses.

I also have several questions regarding the handling of missing data. It is stated that participants with missing smoking or vaping cessation outcomes are imputed as smokers/vapers. It is unclear why this is being referred to as “imputing” as it sounds like participants will be assigned. Why is this being proposed? No rationale is given. I presume to be conservative, assuming that they did not quit. Maybe authors could propose a sensitivity analysis where they are treated differently? Also, is this “assignment” step needed given use of IPW? Why will complete cases results be reported in addition to multiple imputation results?

How will the multiple imputation models be specified? How will you analyze attrition?

The analytic plan to address implementation and maintenance, including cost effectiveness, of the program (objectives 1 iii and iv, key components of the RE-AIM framework guiding the study) are absent in the manuscript text and only briefly described in Table 2. There is no reference to the qualitative aspect of the project that assesses fidelity, barriers, and facilitators. To ensure that all objectives have an analytic plan, consider rewriting the analysis section to indicate which objectives are being met through which analyses. Also, the inclusion of confounding variables (and theoretical justification for each) would be helpful.

Finally, while the authors work hard to demonstrate the collaborative aspects of the study, there are some gaps in the specifics. For instance, the “how” of the community governance and control principles must be better described. Details are needed for the community ethical principles and best practices, as well as data sharing, management, and ownership. What are these practices? Given that an important feature of the study is the co-design with Aboriginal and Torres Strait Islander communities, more information about the important considerations in the specific study context would be a helpful addition to the protocol. Also, there are no coauthors from any of the organizations mentioned as collaborators.

Including these collaborators is an important step in collaborative work.

There are other minor pieces of this manuscript that would benefit from attention. In the abstract, the authors report that there has been no evidence of the effectiveness of this intervention for Aboriginal or Torres Strait Islander people. Given that the authors have already found success with a Type I hybrid effectiveness/implementation study, this language is somewhat misleading.

Language regarding the hybrid study types (I and II) needs to be addressed as well, given that hybrid studies are not a “design”, they are a type of “study.” See: Curran GM, Landes SJ, McBain SA, Pyne JM, Smith JD, Fernandez ME, Chambers DA and Mittman BS (2022) Reflections on 10 years of effectiveness-implementation hybrid studies. Front. Health Serv.

2:1053496. doi: 10.3389/frhs.2022.1053496.

In addition, while the aims are clearly reported on page 7, pieces of them are inconsistently prioritized/referenced throughout the paper. For instance, on page 7 the authors conclude their introduction before moving into the study aims by emphasizing that they will be exploring the cost-effectiveness and geographical uptake differences aspects of their study, which only constitute a small portion of the study, are rarely addressed in the manuscript text, and lack an analysis plan. Please consider ending this section with a broader purpose statement that encompasses both the effectiveness and implementation aims and revising the paper to either 1) remove evaluation specifics and emphasize the effectiveness/implementation goals more generally, or 2) discuss all facets of the aims equally.

Limitations are not discussed, even in the "Strengths and limitations" section of the abstract. A limitations section should be added to the manuscript.

In addition, like the paper, a significant focus of the abstract is on the collaboration with Aboriginal health organizations, limiting space for an adequate description of the methods and analysis plan of the study. Please include a brief analysis plan and measures addressing the key analyses, such as cost-effectiveness, in the abstract.

Finally, greater clarity on terms used throughout the protocol would be helpful. For example, a cut point of 6ppm will be used to define non-smoking. What is ppm? What is "Clothing the Gaps" merchandise? make sure all acronyms are defined (e.g., SF-8D)

VERSION 1 - AUTHOR RESPONSE

Reviewer Comments:
We appreciate the reviewer's thorough and thoughtful feedback on our protocol, and we are grateful for the time and care taken to engage so deeply with the work. We agree that some of the points raised are valid and have helped to strengthen the clarity and rigour of the manuscript.

However, as this submission is a protocol paper for a journal (and not the full protocol for the study), there are practical constraints on the level of methodological and analytical detail that can be included within journal word limits and conventions. In particular, several suggestions related to changing the study design, providing a detailed description of the intervention, providing a detailed statistical analysis plan, and embedding randomized sub-studies are either beyond the scope of a protocol manuscript, or are not feasible within the constraints of the current study design and funding timelines. While we appreciate that some of these suggestions could strengthen the study in an ideal context, applied intervention research necessarily involves balancing methodological rigour with feasibility, ethics, and available resources (both time and money). We note that the current design reflects these considerations and has undergone rigorous peer review through a national competitive funding process, through which the project was awarded substantial funding. Some of the reviewers' comments also appear to be based on misunderstandings of standard reporting conventions in this field, that go beyond the detail required in an academic manuscript. We have clarified sections of our manuscript to better reflect our planned approach while maintaining alignment with standard reporting expectations for

protocol papers. We hope that these revisions strike an appropriate balance between methodological clarity and the requirements of the journal format.

Given the format of the reviewer comments provided and repetition in feedback, we have grouped feedback by issue to address the comments provided.

ABSTRACT

In the abstract, the authors report that there has been no evidence of the effectiveness of this intervention for Aboriginal or Torres Strait Islander people. Given that the authors have already found success with a Type I hybrid effectiveness/implementation study, this language is somewhat misleading.

We agree that the wording may not have sufficiently distinguished between prior evidence and the specific gap this study addresses. We have revised the wording to clarify that, while previous research has demonstrated effectiveness and acceptability of the Which Way? Quit Pack intervention, there is currently no evidence regarding its effectiveness when delivered at a national scale.

In addition, like the paper, a significant focus of the abstract is on the collaboration with Aboriginal health organizations, limiting space for an adequate description of the methods and analysis plan of the study. Please include a brief analysis plan and measures addressing the key analyses, such as cost-effectiveness, in the abstract.

The abstract already includes information on the study design, intervention components, primary outcomes, and use of the RE-AIM framework to evaluate effectiveness and implementation outcomes, including cost-effectiveness. To further clarify the planned analyses, we have added the following two sentences to the Methods and Analysis section of the abstract: "An intention-to-treat approach will be used for cessation outcomes, with participants lost to follow-up classified as smokers/vapers. Exploratory logistic regression analyses will examine factors associated with quitting, and causal machine learning methods will be used to explore variation in intervention effectiveness across participant subgroups." These additions provide a brief overview of the key analytical approaches while remaining consistent with abstract word limits and reporting conventions for protocol manuscripts.

STUDY DESIGN

Most prominent is the single-group study design, specifically the lack of a comparison group, which severely limits the evaluation of the effectiveness of the intervention….The single-group study design limits rigor and the ability to answer the research question. A single-group design can show change over time, but not causal effectiveness. The design is especially limited if the study is meant to assess both effectiveness and implementation, because implementation success can be mistaken for intervention effectiveness when there is no comparison condition. The single group design is a reasonable pilot or early-stage design, but not a strong evaluation design. A community- or service-level stepped-wedge cluster randomized trial with embedded implementation evaluation would be a stronger choice. Alternatively, the authors may consider a wait-list randomized design, which can address ethical concerns around providing all participants who want to quit with support but

is less useful if contamination is likely. Or consider a cluster randomized rollout by service or community. The balance between scientific rigor and ethical considerations needs to be more carefully considered and justified regardless of study design, but especially if the authors choose to retain the single-group study design. For instance, the authors indicate the study design was chosen “in recognition of the challenges of implementing high quality controlled trials in Indigenous context” (p. 10). Rephrasing this sentence to include a few of the challenges that are being addressed through this methodology would better explain the connection.
We acknowledge that the single-group design limits the ability to attribute observed changes directly to the intervention, and we agree that the absence of a comparison group is a methodological limitation. The chosen study design reflects a range of practical, ethical, and contextual considerations that were central to the study design and reflect the realities of conducting applied intervention research in an Aboriginal and Torres Strait Islander context. Importantly, smoking cessation research with Indigenous populations globally has consistently faced substantial methodological challenges, and even randomised controlled trials have faced recruitment and retention difficulties, contamination, implementation challenges, and risks of bias (see Choi 2016, Dignan 2019, Dsilva 2011, Eades 2012, Holt 2005, Marley 2014, Patten 2020, Smith 2014). We have explicitly acknowledged the study design as a limitation in the limitations section of the manuscript.

Refs: Choi 2016 DOI: 10.1016/j.amepre.2016.05.021; Dignan 2019 DOI:10.1016/j.cct.2019.04.007; Dsilva 2011 DOI:10.4278/ajhp.100611-QUAN-180; Eades 2012: DOI:10.5694/mja11.10858; Holt 2005 DOI:10.1136/thx.2004.030239; Marley 2014 DOI:10.1186/1471-2458-14-32; Patten 2020: DOI:10.3390/ijerph17249302; Smith 2014 DOI:10.1177/0011000014542601.

MEASURES
The measures for the study need more explanation. I agree that intervention reach, including participation rates and representativeness, and intervention adoption measured as uptake and use of intervention components are intervention outcome measures. However, the number and proportion of participants completing baseline and follow-up surveys is not an implementation outcome measure (though important to track to assess and describe research study and data quality). Similarly, the proposed measure of implementation maintenance (i.e., smoking and vaping abstinence at 12-month follow-up) is not a measure of implementation sustainment but effectiveness sustainment. I recommend considering a different measure that assesses continued implementation of the intervention (e.g., the proportion of clinics/services still delivering the intervention with acceptable fidelity after active implementation support ends). In RE-AIM, maintenance is defined at the setting level as whether a program becomes part of routine organizational practice, not sustained clinical effectiveness.
We disagree with the reviewer’s interpretation of how maintenance is conceptualised in RE-AIM. Specifically, “In the RE-AIM framework, maintenance of an intervention occurs at two levels: maintenance of individual-level of effects of an intervention (i.e., long-term

effectiveness), and maintenance of the intervention at the setting-level (i.e., long-term implementation)"- see https://re-aim.org/assessing-maintenance/. In the context of this study, the intervention is delivered centrally by our research team rather than through clinics or services. As this limits our ability to apply traditional setting-level maintenance measures, we have conceptualised maintenance to include both sustained individual-level outcomes (e.g., smoking and vaping abstinence at 12 months), and cost-effectiveness as an indicator of program-level sustainability. The rationale for this is already outlined in the text "For this trial, maintenance outcomes have been conceptualised to include individual-level cessation outcomes, reflecting the importance of sustained abstinence and ongoing quit attempts as key indicators of long-term intervention impact in smoking and vaping cessation. Maintenance will therefore be determined by prolonged cessation and quit attempts at 12-months follow-up, as well as an assessment of the cost effectiveness of the intervention as an indicator of program-level sustainability." We have now added a reference for this approach to the paper (https://pmc.ncbi.nlm.nih.gov/articles/PMC8327549/).

I also have questions about participant-reported behaviors. Participants will be asked to provide the number of times they have made a quit smoking attempt lasting longer than 24 hours in the last six months. Will participants be able to answer this accurately? Do the authors have information from prior work that this works?
Participants will be asked to report quit attempts lasting at least 24 hours over relatively short recall periods (three months at the 3- and 6-month follow-ups, and six months at the 12-month follow-up). Self-reported quit attempts over a defined recall period is a widely used measure in smoking cessation research, and subject to the same degree of recall bias as any self-reported behavioural measure. Importantly, the purpose of this measure is not to obtain a precise count of every quit attempt, but rather to capture participants' engagement in cessation behaviour and provide an indication of ongoing efforts to quit. Our previous work using the same question have not identified any major issues with participant comprehension or response patterns, and this approach is considered appropriate for studies of this scale and design.

Since biomedical verification is voluntary and there is no comparison group, this measure is likely to be biased due to self-selection. I suspect that people who quit will be more likely to agree to biomedical verification than those who relapsed. Although biochemical validation is a strength of the study, the study design will likely not provide useful data. Could you design a small randomized sub-study instead to test this, especially since the biochemical verification of smoking status has not previously been piloted in this intervention setting?
We agree that relying on voluntary biochemical verification introduces the potential for self-selection bias, with participants who have successfully quit potentially more likely to opt into verification than those who have relapsed. We have now acknowledged this as a limitation in the manuscript, including in the 'Strengths and Limitations' section at the end of the abstract- "The use of self-reported cessation as the primary outcome may introduce recall and social desirability bias."

Biochemical verification is included in this study as a secondary outcome to strengthen

confidence in self-reported cessation outcomes where feasible; however, the primary outcomes are based on self-report since there is very little evidence in the Australian setting about the feasibility of biochemical verification of self-reported cessation with Indigenous participants. This is already stated in the manuscript "As biochemical verification of smoking status has not previously been piloted in this intervention setting, data generated through this process will also inform adoption and implementation outcomes, including feasibility, acceptability, and participant adherence to verification procedures. These findings will support refinement of verification processes for use in future trials with Aboriginal and Torres Strait Islander people." While incorporating a randomized sub-study could provide additional data about self-selection effects, it is not feasible within the scope, timelines, and funding of the current project. More importantly, research involving Aboriginal and Torres Strait Islander peoples must be conducted in accordance with principles of Indigenous data sovereignty, community partnership, cultural safety, and respect for participant autonomy. Randomising participants to different verification procedures solely to investigate self-selection effects would place additional participant burden and would require extensive consultation and co-design to ensure cultural appropriateness and community acceptability. Such an approach was not supported through the study's governance and community engagement processes.

Attention to intervention cost in the setup of the study is a strength, but the assessment and analysis of cost effectiveness are not well described. What kind of cost data will be collected and how? How will this be analyzed? Why is there a focus on cost effectiveness versus other cost analysis? This is not well justified or described.
Further detail on cost-effectiveness analysis is provided in the manuscript (see page 16). Cost-effectiveness will examine value for money. Using a utility measure such as QALY enables comparison with other health related interventions.

The authors propose to measure implementation fidelity, quality, and consistency only with qualitative measures. There seems to be a missed opportunity for quantitative measurement as well.
Our evaluation of fidelity, quality, and consistency is not solely qualitative, but is supported by multiple quantitative indicators across the RE-AIM framework. As outlined in Table 2, quantitative data are collected on uptake, reach, participant representativeness, and use of intervention components (e.g., NRT use, behavioural counselling engagement, and online forum participation), which provide objective indicators of implementation delivery and consistency. While we acknowledge that within the implementation domain specifically qualitative measures are used, this is complementary to the quantitative data and consistent with best practice in implementation science for identifying barriers and facilitators that cannot be captured through quantitative measures alone.

STUDY AIMS AND OBJECTIVES
The objectives of this study are to evaluate the effectiveness and implementation of the smoking and vaping intervention. Given prior evidence for the effectiveness of the

intervention, stronger justification for why more effectiveness data are needed, specifically a Type II study.
This study substantially increases the sample size (2,000 participants) and follow-up duration (12 months) compared to our earlier work, enabling more robust and longer-term assessment of outcomes, which are critical in smoking and vaping cessation research. The current trial also involves national implementation, providing an opportunity to assess whether previously observed effects are sustained when the intervention is delivered at scale. There is limited evidence on the effectiveness of smoking and vaping cessation interventions specifically for Aboriginal and Torres Strait Islander people at a national level, particularly in the context of vaping. As such, further effectiveness data remain important to confirm generalisability and inform policy and practice. This information is already contained in the introduction section of the manuscript (page 6, lines 4-10).

In addition, while the aims are clearly reported on page 7, pieces of them are inconsistently prioritized/referenced throughout the paper. For instance, on page 7 the authors conclude their introduction before moving into the study aims by emphasizing that they will be exploring the cost-effectiveness and geographical uptake differences aspects of their study, which only constitute a small portion of the study, are rarely addressed in the manuscript text, and lack an analysis plan. Please consider ending this section with a broader purpose statement that encompasses both the effectiveness and implementation aims and revising the paper to either 1) remove evaluation specifics and emphasize the effectiveness/implementation goals more generally, or 2) discuss all facets of the aims equally.
We agree with the reviewer that this detail isn't necessary at the end of the introduction and so have removed this text and instead ended with a broader purpose statement.

INTERVENTION
Some aspects of the intervention could be explained in greater detail. Participants will receive a comprehensive information booklet, which includes details on the additional supports on offer. What are those additional supports?
We have added additional detail to the intervention section of the methods (pages 11 and 12).

The authors claim the intervention provides culturally grounded support, but do not explain what that means in the context of this specific study and population. Please explain and maybe provide a few examples.
Examples are already included.
We believe this is already clearly outlined in our intervention section. Specific examples include:
• "Each Quit Pack will include: (i) Clothing the Gaps merchandise to celebrate pride in culture"
• "A written health education story book developed specifically by the Indigenous Which Way? team that offers culturally relevant advice and guidance on quitting and NRT use"
• "Participants will receive a comprehensive information booklet in their packs which

outlines an introduction to the Aboriginal and Torres Strait Islander Which Way? team members including photos, details on the additional supports on offer and processes for contacting the team and reminding the details of the research process (i.e. follow up timepoints)."

• "Participants will have access to unlimited phone-based behavioural counselling during business hours (Monday-Friday, 9am-5pm) delivered by one of four Aboriginal and Torres Strait Islander team members (men and women)."

• "All participants will have access to online information and support to assist their quit attempt. Information content will be hosted on the Which Way? website and will include educational content and downloadable resources tailored to support Aboriginal and Torres Strait Islander people in their smoking and vaping cessation journeys that have been co-designed as part of previous trials, and interactive tools to assist with goal setting, tracking progress and staying motivated."

There are some gaps in the sample recruitment, treatment assignment, and data collection procedures that would offer greater insight into the study. First, more details about social media recruitment would be useful. For example, what are hierarchical advertising structures? How do algorithms target, that is, based on what? What are organic posts? Where are they placed, on what social media platforms?
Detailed information is already provided on the social media recruitment strategy (page 10, lines 11-19). We state that paid advertisements will be placed using meta for business and have added that this means Facebook and Instagram. We do not agree it is necessary to provide details about the hierarchical advertising structures.

Also, three participant recruitment strategies are proposed, informed by previous pilot studies. Given that data from prior work is available, please provide justification for the use of all three, drawing on previous studies and literature.
The rationale for the three recruitment strategies is described in the manuscript and is directly informed by our prior pilot work, which demonstrated that these approaches were feasible and effective, achieving recruitment rates of 50–200 participants per month (page 10, lines 4–5). Given this, we do not consider additional justification to be necessary and it is unclear what the reviewer is requesting us to add here. The use of multiple recruitment strategies is standard practice in large-scale, community-based research to ensure adequate reach and inclusivity.

Third, how will medical conditions be confirmed to recommend the right NRT? Are a doctor's evaluation and prescription needed? If not, why not?
NRT is available over the counter in Australia and can be commonly accessed at supermarkets and pharmacies without the need for medical supervision. We have added this to the description of the Quit Pack for clarity. A doctor's evaluation is not required prior to use and there are few contraindications for use (the ones that do exist are already outlined in our eligibility criteria- 'Individuals will be deemed ineligible if they are currently enrolled in any other smoking or vaping cessation program, self-report a previous allergy or hypersensitivity to nicotine or any ingredient in NRT products, or report experiencing any

health condition in the six weeks prior to enrolment that is a contraindication for NRT use. This includes heart attack, unstable angina, irregular heart rhythm, stroke/mini-stroke, brain aneurysm or vascular malformations'.

The NRT options seem complex. Are the participants selecting a treatment option, or the researchers? If the participants choose, how will people decide what is best for them? Especially between the high dose and low dose options. Will the study provide a recommendation?
This detail is already provided in the manuscript. As outlined in Figure 1, there are four NRT pack options. We state on page 11, line 15, that participants will be able to select "select one Quit Pack NRT box based on their NRT preferences including combination NRT options, oral NRT only, or patch only". This is an individual preference-based decision. We then state on line 20 "Each participant will receive a high dose or low dose pack, determined by their self-reported baseline smoking and vaping behaviours". Table one is provided specifically to make the choices and dosage of the packs available clear and transparent. The process reflects standard practice in smoking cessation interventions, where treatment selection incorporates both participant preference for NRT type, and behaviour-informed dosing guidance.

Finally, the data collection procedure for the implementation aim of the study is absent. For instance, the qualitative process evaluation using staff feedback is described nowhere in the protocol text, leaving questions about the sample and recruitment (Who counts as project staff? How are/will they be trained?), data collection procedures (How will/what data be documented? What questions will be asked, if any? What is the frequency and duration of the review meetings?), analysis plans (How will fidelity, quality, and consistency be evaluated with a qualitative approach?), and ethical considerations unaddressed.
Additional detail has been added under the implementation outcomes section to address data collection procedures for the implementation outcomes. Implementation measures are also summarised in Table 2. We hope these revisions ensure that sufficient detail is now provided in the protocol text to allow readers to understand the data collection procedures for implementation outcomes.

ANALYSIS
The analysis section is poorly organized, described, justified, and linked to the objectives of the study. What is the primary analysis for the effectiveness question? And what are the primary statistical analyses/models? Will there be pre-post analyses?
Since the primary outcome is 7-day point prevalence at the various time points, as stated the primary analysis is the estimation of the proportion with a 95% CI ("The Reach, Effectiveness, Adoption, Implementation, and Maintenance components will be described as counts and percentages with 95% confidence intervals"). There is no pre-post analysis, since none of the participants are abstinent at baseline (inclusion criteria - smoke or vape daily), a pre-post analysis is not applicable. As stated, further investigation for associations between the quit outcomes and characteristics are via logistic regression.

Second, in the sample size justification, a range of 5% to 50% is used. That is a very big range. This needs to be more clearly justified as a useful range. What are expected cessation numbers?
This range was chosen to reflect that fact the chosen sample size is sufficient to estimate all but the most extreme prevalences (<5%, or since the relationship is symmetric, > 95% prevalence), with very small MOE (maximum of 2% if the proportion if 50%). 7-day point prevalence at 3- and 6-months in the pilot was 23% and 16% respectively. This prevalence estimate from the pilot has been added to the main text.

Since there is no comparison group, how will we know that effects are really due to the intervention, and which component? Since there are so many components, they need to be explored - how do we know it's NRT or counseling etc.?
We agree that in the absence of a comparison group, the study cannot definitively attribute observed effects to the intervention, nor isolate the relative contribution of individual components (e.g., NRT versus counselling). We have acknowledged this as a limitation in the manuscript. However, the primary aim of this study is to evaluate the effectiveness and implementation of a multi-component intervention package as delivered in real-world settings, rather than to dismantle and test the efficacy of each individual component. Determining the independent effects of each element would require a substantially different study design (e.g., factorial or multi-arm randomised trials), which is beyond the scope, feasibility, and objectives of the current project.

No rationale is given for the inverse probability of treatment weighting; please provide, along with a definition of "intention to treat."
The intention-to-treat (ITT) principle refers to analysing participants in the groups to which they were originally assigned or classified, regardless of their level of adherence to the intervention or withdrawal from the study. In smoking and vaping cessation research, this approach is standard and conservative, as participants lost to follow-up are typically assumed to have not achieved cessation. This minimises the risk of overestimating intervention effects due to attrition. This is stated in the manuscript "An Intention to Treat (ITT) analysis will be performed for all effectiveness cessation outcomes, where participants with missing smoking or vaping cessation outcomes are imputed as smokers/vapers." The inverse probability of treatment weighting approach has been removed. All associations between demographic characteristics and the outcomes are exploratory only. This has been corrected in the protocol text.

Since subgroup analyses are proposed, the power analyses need to include power estimates for these secondary analyses.
The study has been appropriately powered for the primary outcome, which is consistent with standard practice. It is not typical or expected to provide formal power calculations for all planned subgroup or secondary analyses, as these are generally considered exploratory and hypothesis-generating rather than confirmatory.

I also have several questions regarding the handling of missing data. It is stated that

participants with missing smoking or vaping cessation outcomes are imputed as smokers/vapers. It is unclear why this is being referred to as "imputing" as it sounds like participants will be assigned. Why is this being proposed? No rationale is given. I presume to be conservative, assuming that they did not quit. Maybe authors could propose a sensitivity analysis where they are treated differently? Also, is this "assignment" step needed given use of IPW? Why will complete cases results be reported in addition to multiple imputation results?
Coding participants with missing smoking or vaping cessation outcomes as continuing smokers/vapers is a standard and widely accepted approach in smoking cessation trials. This approach is commonly referred to as a conservative imputation aligned with the intention-to-treat principle, whereby participants lost to follow-up are assumed to have not achieved cessation. This method is recommended in the cessation literature and is routinely applied in smoking cessation trials to minimise the risk of overestimating quit rates due to differential attrition. We already state that complete case results will also be reported.

With respect to additional analyses and multiple imputation addresses potential bias arising from missing data under different assumptions. Reporting complete case analyses alongside these approaches is intended to provide transparency and allow comparison across analytic methods, which is consistent with good practice in handling missing data. These approaches are complementary.

IPW was not a method being applied to the primary outcome or to handle missing data. Initially it was included to adjust for confounding of the relationship between demographic characteristics and the outcomes in the secondary analyses, however as these are purely exploratory this has now been removed from the protocol.

How will the multiple imputation models be specified?
Multiple imputation will be by MICE (multiple imputed chained equations), including all factors in the analytic models, and any factors theoretically assumed to be predictive of the missing data. This has been added to the methods.

How will you analyze attrition?
Attrition will be reported in the participant flow chart, and the baseline demographic characteristics of completers and non-completers will be compared via t-tests and chi-squared tests as appropriate.

The analytic plan to address implementation and maintenance, including cost effectiveness, of the program (objectives 1 iii and iv, key components of the RE-AIM framework guiding the study) are absent in the manuscript text and only briefly described in Table 2. There is no reference to the qualitative aspect of the project that assesses fidelity, barriers, and facilitators. To ensure that all objectives have an analytic plan, consider rewriting the analysis section to indicate which objectives are being met through which analyses. Also, the inclusion of confounding variables (and theoretical justification for each) would be helpful.
Additional information has been added to the methods about the cost effectiveness analysis

and collection of the qualitative data. Secondary analyses are now restricted to exploratory univariable analyses, and thus reference to confounders is no longer required.

GOVERNANCE

Finally, while the authors work hard to demonstrate the collaborative aspects of the study, there are some gaps in the specifics. For instance, the "how" of the community governance and control principles must be better described. Details are needed for the community ethical principles and best practices, as well as data sharing, management, and ownership. What are these practices? Given that an important feature of the study is the codesign with Aboriginal and Torres Strait Islander communities, more information about the important considerations in the specific study context would be a helpful addition to the protocol.

We have revised the 'Community Governance and Control' section to clarify how these principles are operationalised in practice. Specifically, we have clarified the role of the Governance Committee that comprises NACCHO, AH&MRC, VACCHO, Aboriginal and Torres Strait Islander investigators and Community Researchers as holding oversight and decision-making authority across all stages of the research, including design, implementation, data management, analysis, and dissemination.

There are no coauthors from any of the organizations mentioned as collaborators. Including these collaborators is an important step in collaborative work.

Representatives from our collaborating organisations were invited to be co-authors on this manuscript and were given the opportunity to review and contribute to its development. However, they elected not to be listed as co-authors and instead preferred their contributions to be recognised in the acknowledgements. We have therefore acknowledged these organisations and their contributions in accordance with their preferences and accepted authorship guidelines

Language regarding the hybrid study types (I and II) needs to be addressed as well, given that hybrid studies are not a "design", they are a type of "study." See: Curran GM, Landes SJ, McBain SA, Pyne JM, Smith JD, Fernandez ME, Chambers DA and Mittman BS (2022) Reflections on 10 years of effectiveness-implementation hybrid studies. Front. Health Serv. 2:1053496. doi: 10.3389/frhs.2022.1053496.

Design has been changed to study in the two relevant places and the title.

Limitations are not discussed, even in the "Strengths and limitations" section of the abstract. A limitations section should be added to the manuscript.

The strengths and limitations section of the abstract has been reviewed in line with the editors' comments. A strengths and limitations paragraph has been added to the discussion.

Finally, greater clarity on terms used throughout the protocol would be helpful. For example, a cut point of 6ppm will be used to define non-smoking. What is ppm? What is "Clothing the Gaps" merchandise? make sure all acronyms are defined (e.g., SF-8D)

We have clarified terms and abbreviations used throughout.

VERSION 2 - REVIEW

**Reviewer**	**1**
**Name**	**Oesterle, Sabrina**
**Affiliation**	**Arizona State University, School of Social Work**
**Date**	**22-Jul-2026**
**COI**	

This protocol describes an important national implementation study addressing smoking cessation among Aboriginal and Torres Strait Islander peoples. The intervention builds on previous pilot and hybrid implementation studies, the study is clearly well-developed in partnership with Indigenous communities, and the manuscript addresses an important public health issue. I continue to have several concerns regarding the clarity with which the study aims, methods, and limitations are described. My comments primarily relate to scientific transparency rather than the underlying study itself.

Major concerns

1. Clarify the interpretation of "effectiveness" within the context of the study design

My principal remaining concern relates to the way the manuscript frames the effectiveness component of this single-arm hybrid Type II effectiveness-implementation study. I appreciate that this study builds on previous pilot and Type I hybrid studies and represents the next stage of evaluating the intervention at national scale. However, throughout the manuscript, effectiveness is often presented as a primary objective without sufficiently explaining what is meant by "effectiveness" in the context of an uncontrolled implementation study. For example, statements such as: "This study will utilise a pragmatic hybrid Type II effectiveness-implementation study to evaluate the effectiveness of the intervention while also gathering data to inform sustainable national implementation," may imply that establishing intervention effectiveness is the primary objective of the present study. Given the absence of a comparison group, I encourage the authors to frame the study more explicitly as building on previous evidence of effectiveness while evaluating implementation at national scale and concurrently assessing real-world effectiveness outcomes. A more appropriate way of describing the study aims could be, for example: This Type II hybrid study evaluates implementation while examining whether the intervention achieves comparable outcomes when delivered at scale. It would also be useful to acknowledge early on that because the study is an uncontrolled national implementation study, observed abstinence rates should be interpreted as real-world implementation outcomes and not as definitive evidence of causal intervention effectiveness.

Similarly, I encourage the authors to distinguish more clearly between the RE-AIM framework and the study design. RE-AIM provides a framework for evaluating implementation and effectiveness outcomes, whereas the study design determines the strength of inference that can be drawn regarding intervention effectiveness. At present, the manuscript occasionally appears to conflate the framework used to categorize outcomes with the methodological basis for evaluating those outcomes. Making this distinction explicit would help readers outside implementation science better understand both the purpose of the effectiveness evaluation and the conclusions that the study is designed to support.

2. Expand the discussion of study limitations

The limitations section currently identifies several important limitations but generally does not explain how these limitations influence interpretation of the findings.

The limitation of the single-arm design is acknowledged in a single sentence. I encourage the authors to briefly explain what this means for interpretation of the effectiveness outcomes. For example, observed abstinence rates should be interpreted as estimates of real-world outcomes achieved during implementation rather than definitive evidence of causal intervention effectiveness.

Reliance on self-reported smoking outcomes is identified as a limitation, but the manuscript does not explain how this may affect interpretation of the findings (e.g., by influencing the accuracy of estimated abstinence rates).

Similarly, limited assessment of intervention fidelity, adherence, and the relative contribution of individual intervention components is noted, but the implications are not described. Readers would benefit from understanding how these limitations affect interpretation of the effectiveness outcomes and the ability to identify which intervention components contribute to successful implementation.

These additions need only be brief but would substantially improve interpretation of the study.

3. Describe the planned analyses for implementation outcomes

The manuscript now provides a statistical analysis plan for the effectiveness and cost-effectiveness outcomes. However, although implementation outcomes and associated measures are described, the manuscript does not describe how these outcomes will be analyzed.

Given that this is presented as a hybrid Type II effectiveness-implementation study, I encourage the authors to include a brief description of the planned analyses for the implementation outcomes (e.g., quantitative analyses, qualitative analyses where applicable, and any planned integration of implementation and effectiveness findings). At present, the manuscript describes what implementation data will be collected but not how those data will address the implementation objectives.

A similar imbalance is evident in the abstract, where the effectiveness and cost-effectiveness analyses are summarized but the planned implementation analyses are not. A brief summary of the implementation analytic approach would better reflect the hybrid nature of the study.

4. Improve clarity of several key methodological descriptions

My remaining comments relate to improving clarity rather than requesting additional methodological detail.

Several important concepts are introduced but remain insufficiently explained for readers who may not be familiar with implementation science, smoking cessation research, or the Australian context.

Culturally relevant intervention content. Because cultural adaptation is presented as a defining feature of the intervention, I encourage the authors to provide two or three brief examples of what is meant by "culturally relevant" or “culturally grounded” content. This is not a request for a detailed intervention manual, but rather illustrative examples that help readers understand how the intervention differs from standard smoking cessation support. For example, the intervention may have incorporated Aboriginal artwork and imagery; advice acknowledged the role of family and community support; content addressed cultural and historical factors influencing smoking; or examples and language reflected community preferences.

Digital recruitment methods. Terms such as "hierarchical advertising structures" and "targeted algorithms" are specialized terminology that may not be familiar to the journal's readership. I recommend either briefly defining these terms or replacing them with simpler language describing the planned recruitment strategy.

Participant selection of NRT. Although the manuscript outlines the available NRT options, I remain uncertain how participants will be supported to select an appropriate product and dose in practice. My concern is not with participant preference itself, but with how this is operationalized. I encourage the authors to briefly describe what decision support participants receive (e.g., automated recommendations, dosing guidance), or alternatively state that this participant-led approach has been successfully implemented in previous studies of this intervention.

Missing smoking data. I appreciate that classifying participants with missing smoking outcomes as smokers is standard practice in smoking cessation research. My comment is not intended to challenge this approach but to encourage the authors to briefly explain the rationale, cite the relevant methodological guidance, and use terminology such as "classified as smokers" rather than "imputed as smokers."

Minor concerns

I appreciate the additional clarification regarding the attrition analyses provided in the response letter. However, these details do not currently appear in the manuscript. Because

these analyses are prespecified, I encourage the authors to incorporate them into the protocol rather than leaving them only in the response letter. Similarly, the response letter explains that complete-case analyses and multiple imputation will both be presented to improve transparency. This rationale would also be helpful in the manuscript.

The current attrition analysis plan compares demographic characteristics of completers and non-completers. Given that attrition in smoking cessation studies may also be related to smoking severity, quit history, early intervention engagement, or smoking outcomes during follow-up, I encourage the authors to prespecify analyses examining predictors of attrition beyond baseline demographics alone. As interim follow-up assessments are planned, consideration should also be given to examining whether earlier smoking status or other key study outcomes are associated with subsequent loss to follow-up.

The response letter explains why organizational-level maintenance is not assessed (because the intervention is delivered centrally rather than through individual clinics). This explanation would strengthen the manuscript and help readers understand why the Maintenance dimension focuses on participant outcomes rather than organizational sustainment.

The response letter also notes that previous work found no major issues with participant recall of quit attempts. Including this information (with an appropriate citation, if available) would help justify the use of this measure.

Please ensure that all abbreviations are defined at first use throughout the manuscript.

VERSION 2 - AUTHOR RESPONSE

Reviewer Comments: 1

1. Clarify the interpretation of "effectiveness" within the context of the study design

My principal remaining concern relates to the way the manuscript frames the effectiveness component of this single-arm hybrid Type II effectiveness-implementation study. I appreciate that this study builds on previous pilot and Type I hybrid studies and represents the next stage of evaluating the intervention at national scale. However, throughout the manuscript, effectiveness is often presented as a primary objective without sufficiently explaining what is meant by "effectiveness" in the context of an uncontrolled implementation study. For example, statements such as: "This study will utilise a pragmatic hybrid Type II effectiveness-implementation study to evaluate the effectiveness of the intervention while also gathering data to inform sustainable national implementation," may imply that establishing intervention effectiveness is the primary objective of the present study. Given the absence of a comparison group, I encourage the authors to frame the study more explicitly as building on previous evidence of effectiveness while evaluating implementation at national scale and concurrently assessing real-world effectiveness outcomes. A more appropriate way of describing the study aims could be, for example: This Type II hybrid study evaluates implementation while examining whether the intervention achieves comparable outcomes when delivered at scale. It would also be useful to acknowledge early on that because the study is an uncontrolled national implementation study, observed abstinence rates should be

interpreted as real-world implementation outcomes and not as definitive evidence of causal intervention effectiveness.
We agree that the current study does not permit causal inference regarding intervention effectiveness. However, consistent with hybrid Type II effectiveness-implementation designs (see https://pmc.ncbi.nlm.nih.gov/articles/PMC6779135/#F1), the study includes both implementation and effectiveness outcomes as co-primary outcomes. We have revised the manuscript to clarify that the effectiveness component relates to evaluating participant quit outcomes achieved during national-scale implementation.

See edits on page 9 to now read: "This study will utilise a pragmatic hybrid Type 2 effectiveness-implementation design to examine the impact of the Which Way? Quit Pack intervention on individual cessation outcomes, while also gathering data to evaluate the implementation and inform sustainable national implementation."

Similarly, I encourage the authors to distinguish more clearly between the RE-AIM framework and the study design. RE-AIM provides a framework for evaluating implementation and effectiveness outcomes, whereas the study design determines the strength of inference that can be drawn regarding intervention effectiveness. At present, the manuscript occasionally appears to conflate the framework used to categorize outcomes with the methodological basis for evaluating those outcomes. Making this distinction explicit would help readers outside implementation science better understand both the purpose of the effectiveness evaluation and the conclusions that the study is designed to support.
This has now been re-worded in the manuscript for clarity (pg. 6 lines 15-17, pg. 9 lines 12-13).

2. Expand the discussion of study limitations
The limitations section currently identifies several important limitations but generally does not explain how these limitations influence interpretation of the findings.
The limitation of the single-arm design is acknowledged in a single sentence. I encourage the authors to briefly explain what this means for interpretation of the effectiveness outcomes. For example, observed abstinence rates should be interpreted as estimates of real-world outcomes achieved during implementation rather than definitive evidence of causal intervention effectiveness.
Reliance on self-reported smoking outcomes is identified as a limitation, but the manuscript does not explain how this may affect interpretation of the findings (e.g., by influencing the accuracy of estimated abstinence rates).
Similarly, limited assessment of intervention fidelity, adherence, and the relative contribution of individual intervention components is noted, but the implications are not described. Readers would benefit from understanding how these limitations affect interpretation of the effectiveness outcomes and the ability to identify which intervention components contribute to successful implementation.
These additions need only be brief but would substantially improve interpretation of the study.
The limitations section (page 17) has now been updated per reviewer comments.

3. Describe the planned analyses for implementation outcomes

The manuscript now provides a statistical analysis plan for the effectiveness and cost-effectiveness outcomes. However, although implementation outcomes and associated measures are described, the manuscript does not describe how these outcomes will be analyzed. Given that this is presented as a hybrid Type II effectiveness-implementation study, I encourage the authors to include a brief description of the planned analyses for the implementation outcomes (e.g., quantitative analyses, qualitative analyses where applicable, and any planned integration of implementation and effectiveness findings). At present, the manuscript describes what implementation data will be collected but not how those data will address the implementation objectives.
While being mindful of wordcount for a protocol manuscript, the wording in the analysis section has been adjusted as follows: "The Reach, Effectiveness, Adoption, Implementation, and Maintenance quantitative components will be described as counts and percentages with 95% confidence intervals and qualitative components will be thematically analysed (see Table 2)." We have added quantitative and qualitative analysis approaches to the items in the table.

A similar imbalance is evident in the abstract, where the effectiveness and cost-effectiveness analyses are summarized but the planned implementation analyses are not. A brief summary of the implementation analytic approach would better reflect the hybrid nature of the study.
Noting the abstract word count limitations which restrict in depth description of all analysis components, this has been updated to read 'The RE-AIM (Reach, Effectiveness, Adoption, Implementation, and Maintenance) framework will guide evaluation of implementation and effectiveness outcomes. Analysis measures include cessation and quit attempt rates, number of participants, representativeness, and intervention uptake.'

4. Improve clarity of several key methodological descriptions
My remaining comments relate to improving clarity rather than requesting additional methodological detail.
Several important concepts are introduced but remain insufficiently explained for readers who may not be familiar with implementation science, smoking cessation research, or the Australian context.
Culturally relevant intervention content. Because cultural adaptation is presented as a defining feature of the intervention, I encourage the authors to provide two or three brief examples of what is meant by "culturally relevant" or "culturally grounded" content. This is not a request for a detailed intervention manual, but rather illustrative examples that help readers understand how the intervention differs from standard smoking cessation support. For example, the intervention may have incorporated Aboriginal artwork and imagery; advice acknowledged the role of family and community support; content addressed cultural and historical factors influencing smoking; or examples and language reflected community preferences
The intervention is culturally grounded rather than adapted, which is an important distinction. We have revised the manuscript throughout to detail this further, particularly page 11 and 12.

Digital recruitment methods. Terms such as "hierarchical advertising structures" and "targeted algorithms" are specialized terminology that may not be familiar to the journal's readership. I recommend either briefly defining these terms or replacing them with simpler

language describing the planned recruitment strategy.
This has been adjusted to read: '(i) paid, targeted advertisements using Meta for Business (Facebook and Instagram posts)' (pg. 10).

Participant selection of NRT. Although the manuscript outlines the available NRT options, I remain uncertain how participants will be supported to select an appropriate product and dose in practice. My concern is not with participant preference itself, but with how this is operationalized. I encourage the authors to briefly describe what decision support participants receive (e.g., automated recommendations, dosing guidance), or alternatively state that this participant-led approach has been successfully implemented in previous studies of this intervention.
This is detailed on page 11 of the manuscript. We have replaced the words 'determined by' with 'based on their' to improve clarity in the fourth sentence. The section now reads:

"Participants will have the opportunity to select one Quit Pack NRT box (see Table 1) based on their NRT preferences, including combination NRT options, oral NRT only, or patch only (for the second mailing only). The staged delivery of NRT is designed to balance clinical effectiveness, participant autonomy, and efficient resource use. Provision of a standardised 8-week baseline supply supports early quit attempts by reducing decision burden at enrolment and ensuring access to evidence-based dosing during the period of highest relapse risk. Each participant will receive either a high dose or low dose pack based on their self-reported baseline smoking and vaping behaviours (see Figure 1). The follow-up 4-week supply allows participants to tailor their NRT based on lived experience of cravings, product acceptability, and adherence, and will be selected by participants during confirmation of additional supply. Dosage of NRT is in line with RACGP guidelines for smoking cessation, and in the absence of specific evidence about dosage of NRT for vaping, the same guidelines will be used to support vaping cessation38"

Missing smoking data. I appreciate that classifying participants with missing smoking outcomes as smokers is standard practice in smoking cessation research. My comment is not intended to challenge this approach but to encourage the authors to briefly explain the rationale, cite the relevant methodological guidance, and use terminology such as "classified as smokers" rather than "imputed as smokers."
This has been updated to 'classified' in the manuscript and a reference has been added.

I appreciate the additional clarification regarding the attrition analyses provided in the response letter. However, these details do not currently appear in the manuscript. Because these analyses are prespecified, I encourage the authors to incorporate them into the protocol rather than leaving them only in the response letter. Similarly, the response letter explains that complete-case analyses and multiple imputation will both be presented to improve transparency. This rationale would also be helpful in the manuscript.
The following has been added to the manuscript 'Attrition will be reported in the participant flow chart, and the baseline demographic characteristics of completers and non-completers will be compared via t-tests and chi-squared tests as appropriate. Complete case results will also be reported to provide transparency and allow comparison across analytic methods.'

(page 16)

The current attrition analysis plan compares demographic characteristics of completers and non-completers. Given that attrition in smoking cessation studies may also be related to smoking severity, quit history, early intervention engagement, or smoking outcomes during follow-up, I encourage the authors to prespecify analyses examining predictors of attrition beyond baseline demographics alone. As interim follow-up assessments are planned, consideration should also be given to examining whether earlier smoking status or other key study outcomes are associated with subsequent loss to follow-up.
Baseline characteristics of completers and non-completers will be compared as appropriate, which is not limited to demographic information alone. This has been added to page 16 of the manuscript.

The response letter explains why organizational-level maintenance is not assessed (because the intervention is delivered centrally rather than through individual clinics). This explanation would strengthen the manuscript and help readers understand why the Maintenance dimension focuses on participant outcomes rather than organizational sustainment.
The authors believe that this is already clear in the manuscript as it is described as a 'mail-out' cessation trial with 'self-refer' detailed in the recruitment strategy. There is no mention of organizations delivering this intervention.

The response letter also notes that previous work found no major issues with participant recall of quit attempts. Including this information (with an appropriate citation, if available) would help justify the use of this measure.
This has been adjusted with a reference added on page 16.

Please ensure that all abbreviations are defined at first use throughout the manuscript.
We have reviewed and believe all abbreviations are defined at first use.
