# Supplementary material for: Which Way? Quit Pack study protocol: a national hybrid type 2 effectiveness-implementation study of mailed smoking and vaping cessation support for Aboriginal and Torres Strait Islander people
Source: BMJ Open. 2026 Sep 11;16(9):e121440. doi: 10.1136/bmjopen-2026-121440 (PMC13583795; doi:10.1136/bmjopen-2026-121440)
Supplement: Supplementary file 2 [file bmjopen-16-9-s001.docx]

**Consent**

Welcome to the Which Way? Quit Pack. Before we get started, we want to ask you a few questions to make sure you understand what’s involved and that it’s safe for you to use NRT.

You can access more details about the research here: (Participant Information Sheet attached)

Once you’ve read the information sheet, you can agree to participate in the research. It is entirely your choice to participate. This means that you can say no.

If you decide to participate, please confirm that you:

1. Understand the purpose of the research and have been given the opportunity to ask any questions.

2.Know that you have the right to withdraw your consent and involvement in the research project at any time without providing a reason.

3. Understand the information you provide during the survey will remain confidential. Your personal details will not be identifiable in any publications arising from the research.

4. Confirm that you are not part of another program to help you quit the smokes or vapes

□ Yes, I agree to take part	□ No, I do not want to take part
